# Increasing jute (*Corchorus olitorius* L.) fiber yield through hybridization and combining ability studies to break the yield plateau

**DOI:** 10.3389/fpls.2025.1499256

**Published:** 2025-02-26

**Authors:** Kumar Nishant Chourasia, Jitendra Kumar Meena, Rakesh Bhowmick, Vikas Mangal, Anil Kumar Arroju, Thribhuvan R, Chandan Sourav Kar, Amit Bera, Pratik Satya, Jiban Mitra, Gouranga Kar

**Affiliations:** ^1^ Division of Crop Improvement, ICAR Central Research Institute for Jute and Allied Fibres, Kolkata, India; ^2^ Division of Crop Improvement, ICAR Central Potato Research Institute, Shimla, India; ^3^ Division of Crop Improvement, ICAR-Indian Institute of Oilseeds Research, Hyderabad, India

**Keywords:** yield barrier, jute, heterosis, combining ability, diversity, molecular markers, sustainable fiber production

## Abstract

The growing global demand and shift from synthetic to natural fibers highlight the need to overcome the yield plateau in jute production. Despite being a sustainable alternative to plastic, jute faces declining cultivation, making yield improvement crucial to meet increasing demand. In this direction, the study was designed to explore hybridization and combining ability to improve the genetic yield potential of jute. Using a diallel mating design, 90 hybrid combinations were evaluated along with 10 parental lines, focusing on traits such as fiber yield, plant height, basal diameter, stick weight, and green biomass. The investigation revealed JROBA 3 and JBO 1 as the most effective general combiners, highlighting their significant potential as parents to produce outstanding hybrids and generate good transgressive segregants. Among the tested hybrids, JROBA 3 × JRO 2407 was found to have very high specific combining ability (SCA), yielding 24.42% more than the national check variety, JRO 204. A correlation study was also conducted, revealing that stick weight had a strong positive correlation with fiber yield, highlighting it as a key factor in selecting high-yielding hybrids. This study also identified the hybrid JROBA 3 x JBO 1 which exhibited an 18% biomass yield advantage over the national check variety. Positive mid-parent heterosis and better-parent heterosis were observed in hybrids, further demonstrating the effectiveness of hybridization in jute breeding. Parent genetic diversity was characterized using intron-length polymorphism markers. Molecular diversity analysis categorized the varieties into two distinct clusters, suggesting possible avenues for integrating improved features into future jute types. This study has established the fact that heterosis breeding can efficiently improve fiber productivity through the involvement of non-additive gene action. The application of heterosis breeding to improve jute production presents a significant opportunity for breeders, aligning well with sustainable development goals and promoting the use of biodegradable fiber alternatives.

## Introduction

Jute (*Corchorus olitorius* L.) is a lignocellulosic fiber and a versatile crop due to its various properties such as being biodegradable, recyclable, and eco-friendly (Mir et al., 2008). It also possesses good strength, high cellulose content, and low microfibril angle. Jute fibers also possess excellent energy absorption capacity and decent fire resistance. Furthermore, the presence of lipids; proteins; vitamins A, C, and E; iron; sodium; phosphorous; calcium; and potassium contributes to its high nutritive value, and, therefore, it is consumed as a leafy vegetable globally (Tareq et al., 2019). These attributes highlight its potential applications in nanomaterials, reinforced cementitious composites, supercapacitors, high-value textiles products, packaging, paper, agriculture, crafts, and bio-fuel, making it essential for industrial and sustainable uses (Song et al., 2021). The fibers are also used in making twines, ropes, and sacks (Adeyemo et al., 2021). Therefore, this is one of the most robust natural fibers and is considered the 'fiber for the future'

Over the last decade, plastic has posed a significant environmental threat. In contrast, jute, being a natural fiber and biodegradable, stands out as one of the most effective alternatives to plastic. The global demand for jute bags is approximately 500 billion units annually (Alimuzzaman et al., 2024). Substituting plastic bags with jute will contribute to the achievement of the sustainable development goals of the nation. India and Bangladesh account for more than 90% of global jute demand and are the primary nations driving jute exports. Saudi Arabia, Syria, Indonesia, the USA, Iran, Sudan, the European Union, Turkey, and China are major importers of jute and its products (CACP, 2021). In the year 2021-22, exports of jute and its products from India and Bangladesh increased by 31%, rising from 370 million USD in 2020-21 to 512 million USD in 2021-22 (CACP,2021). Compared to the previous decade (2010-2020), India's jute cultivation area has declined by approximately 12%. In the 1980s, the average jute yield was approximately 1.6 tons per hectare. This figure rose by about 19% to reach 1.9 tons per hectare, followed by a further increase of 21% to 2.3 tons per hectare in the 2000s. However, the past decade witnessed a modest increase of only 13%. (CACP, 2021). This situation arises from competition with other profitable crops and a reduction in available cultivable area. Given the rising global demand and increasing preference for natural over synthetic fibers, it is essential to overcome the current yield plateau of 25 quintals/hectare to meet the demands of this golden fiber.

Trait variability is crucial for plant breeders aiming to develop improved cultivars, achievable through processes such as mutation, hybridization, chromosome engineering, and genome editing. Among these, hybridization stands out as an incredible technique for generating new gene recombination, allowing for the selection of desirable recombinants that can be advanced to produce stable lines. Jute is predominantly a self-pollinating crop, which limits the economic viability of developing F_1_ hybrid cultivars unless genetic male sterility systems are identified and utilized. Another approach to leverage hybridization is to create favorable gene combinations for which the study of parental lines is crucial. The genetic potential of the parents primarily influences the progenies' performance. Breeders need to identify superior parental lines to overcome yield limitations and enhance genetic improvement in jute. Relying solely on individual performance is insufficient; a comprehensive understanding of gene actions is crucial. In this context, studies on combining ability play a crucial role. The application of a diallel mating system with reciprocals is recommended to improve fiber yield in jute. This enables breeders to compute general and specific combining abilities (GCA and SCA), identify superior parents, and recognize hybrids with improved fiber traits (Zhang et al., 2019). Additional reciprocal crosses assess maternal effects together with cytoplasmic inheritance, both of which are crucial for traits like fiber yield (Griffing, 1956). This approach improves the effectiveness of identifying high-yielding lines and aligns with enhancing efficiency in breeding programs aimed at increasing fiber yield, which is crucial for the economic and industrial applications of jute.

Careful selection and the application of heterosis breeding in jute hold significant promise for breaking through the yield plateau and improving the variety replacement rate (VRR). Hybrid jute cultivars show a 20% to 30% increase in fiber production when compared to conventional varieties (Khatun et al., 2010) In addition, it improves the performance of hybrids in extreme stress conditions, thus enhancing their adaptability (Mba et al., 2012). The introduction of high-yielding varieties acts as a motivation for farmers to adopt new varieties, thus increasing their income (Roy et al., 2020).

The molecular characterization of genotypes facilitates the clear identification and assessment of genetic diversity (Chourasia et al., 2023). Molecular markers such as intron length polymorphism (ILP) have been widely utilized in industrial crops to investigate the diversity of germplasm in hybrid breeding (Li et al., 2013 and Shen et al., 2024). Introns, which are non-coding regions, exist within eukaryotic genomes between exons and experience minimal selective pressure (Jo and Choi, 2015; Kostygov et al., 2024). Previous investigations have indicated that intron sequences evolve at a significantly faster rate and exhibit a higher frequency of polymorphisms compared to exons. These traits position them as valuable polymorphic molecular markers. The application of ILPs in the jute crop improvement program remains extremely limited. Furthermore, it is essential to address the yield plateau in jute. This phenomenon occurs as a result of diminished genetic diversity and the continuous selection of analogous traits across generations (Khatun et al., 2010). Therefore, this study was designed to address this plateau through hybridization and selection, aiming to create new gene combinations that increase fiber yield.

## Material and methods

### Plant material and field-based parameter evaluation

Ten genotypes, selected for traits such as pre-flowering maturity resistance, fine fiber quality, and resistance to biotic and abiotic stresses, were crossed in a diallel design as per Griffing (1956). The details of the parents are given in **Table 1**. A total of 90 crosses, including reciprocals, were developed between August and November 2021 at the breeding station of ICAR-CRIJAF. Between March and Jun 2022, 90 hybrid crosses, 10 parental lines, and a national check variety (JRO 204) were assessed at the Central Research Institute for Jute and Allied Fibres (ICAR-CRIJAF) (Kolkata, India) breeding station. **Supplementary Table S1** contains the list of hybrid crosses.

**Table 1:** Parents used in the study.

S.No.	Advanced breeding lines/variety	Traits
1.	JROBA 3	Interspecific derived line, high-yielding, premature flowering resistance
2.	JROBA 4	Advanced breeding line and high-yielding
3.	JRO 204	Premature flowering resistance
4.	JRO 2407	Premature flowering resistance
5.	S 19	Resistant to premature flowering, tolerant to major pests and diseases, finer fiber quality with lower lignin content.
6.	JBO 1	Low lignin content, resistance to premature flowering, better fiber quality, resistance to major pest and diseases
7.	JROM 1	High-yielding
8.	JROMU 1	Insect and disease tolerant
9.	JRO 524	Resistant to root rot diseases in high rainfall areas
10.	JRO 8432	Premature flowering resistance

A total of 101 test materials (90 crosses, 10 parents, and 1 check) were planted in a randomized complete block design with three replications. Row-to-row spacing was 45 cm, and apart from this all recommended agronomic packages of practice were followed (Sarkar et al., 2013). JRO 204 was planted along the border of the test plot to act as a border crop, thereby minimizing the border effect. The presence of a buffer crop enhances the reliability of the experimental results by creating a buffer zone that stabilizes growth conditions for the plants under study. After 120 days, entries were harvested on a block basis, and retting was conducted using free-flowing water technology developed by ICAR-CRIJAF using CRIJAF Sona™ as the initial inoculum. Yield parameters such as plant height (cm), basal diameter (mm), green weight/plant (g), stick weight/plant (g), and fiber weight/plant (g) were recorded.

### Estimation of heterosis

Mid-parent heterosis (MH), better-parent heterosis (BH), and standard heterosis (SH) were estimated for each hybrid combination according to previously reported studies (GH Liang, 1972; Nadarajan and Gunasekaran, 2005). The mean of the check JRO 204 was used for the estimation of standard heterosis as it was the best-performing national check in the All India Coordinated Research Trial of Jute and Allied Fibres (AINPJAF).

### Combining ability analysis

In total, 90 cross combinations, including reciprocals, were developed from the crossing of 10 parents in a diallel fashion (Jinks, 1953) and were analyzed for their combining ability according to the methods of Griffing (1956) and Singh and Chaudhary (1979). Computation of GCA, SCA, reciprocal effects, and their variance was performed using the 'Agricolae' package (Mendiburu et al., 2015) in R v3.2.3 (R Development Core Team, 2008). Since all the parents used in this study were homozygous and homogeneous, additive (σ^2^_A_) and dominance (σ^2^_D_) genetic variances were calculated assuming inbreeding coefficient (F) = 1. Broad (H^2^) and narrow sense (h^2^) heritability for the measured traits were estimated based on Griffing (Griffing, 1956).

### DNA extraction

Genomic DNA was extracted from all 10 parental lines involved in the hybridization program. The extraction was performed from 100 mg fresh leaf tissues using the CTAB method as outlined by Doyle and Doyle (1987). The tissues were grounded in liquid nitrogen and resuspended in 700 μL of the CTAB extraction buffer (2% CTAB, 100 mM Tris-HCl, 20 mM EDTA, 1.4 M NaCl, 0.2% β-mercaptoethanol) and incubated at 65°C for 30 min. After incubation, the aqueous phase containing DNA was separated using chloroform: isoamyl alcohol (24:1). Then, the DNA was precipitated in chilled isopropanol and washed using 70% ethanol.

### PCR amplification of ILP markers

ILP markers were utilized to assess the genetic diversity among all 10 parents of *C. olitorius*. For the PCR, the total volume of 20 µL contained 2 µL of template DNA (~50 ng/µL); 10 µL of PCR master mix containing Taq polymerase, dNTPs, MgCl2, and reaction buffer; 1 µL of forward primer (10 µM); 1 µL of reverse primer (10 µM); and 6 µL of nuclease-free water. The following amplification condition was used in the thermal cycler (Applied Biosystems, USA): an initial denaturation step of 5 min at 94°C, followed by 35 cycles of 45s at 94°C, 1 min at 52°C to 61°C, and 3 min at 72°C with a final extension at 72°C for 7 min. The PCR products were separated on a 2% agarose gel followed by visualization under UV illumination (Vilber Gel Documentation System, France)

### Data analysis and construction of dendrogram

The bands of the amplified ILP markers were scored as binary data (i.e., ‘1’ for presence, ‘0’ for absence). Nei’s genetic similarity index (Nei, 1972) was determined. The matrix showing the genetic similarity was analyzed by NTsysPC software v2.1. A dendrogram was constructed using the UPGMA based on the accession genetic relationships.

### Statistical analyses

A general linear model built in SAS version 9.4 (SAS Institute, Cary, NC, USA) was used for the analysis of variance (ANOVA). For mean comparisons among genotypes, we used Tukey’s HSD test (*post hoc* test between genotypes, P < 0.05 and P < 0.01). Correlation among traits was computed based on Pearson’s product-moment correlation as implemented in R (R Development Core Team, 2008). All analyses were done with the help of IndoStat Software (Indostat, Hyderabad, India) and further cross-checked with the TNAUSTAT statistical package (Manivannan, 2014). The significance of heterosis was done via a t-test (P < 0.05 and P < 0.01) as described by Turner (1953).

## Results

### Parent and hybrid performance

Based on the results of the ANOVA, significant genotype effects for all characterized traits were found. The examined traits' coefficients of variation (CVs) were all less than 20%, demonstrating adequate and legitimate experimental accuracy (**Table 2**). The mean performance of the F_1_ progenies and parental lines for fiber yield and yield components are presented in **Table 3**. Plant height ranged from 401.3 cm in the hybrid JROBA 4 × JROM1 to 498.93 cm in the hybrid JBO 1 × S 19, with an average of 443.53 cm The average basal diameter recorded among the hybrids and parents was 19.48 mm. It varied from 16.52 mm (JRO 204 × S 19) to 24.39 mm (JRO 524 × JROM 1). The average fiber weight/plant of the hybrids was significantly higher than that of the parents. All the top fiber yielders were hybrids (JROBA 3 × JRO 2407, JRO 8432 × JROM 1, JROBA 3 × JRO 8432, JROBA 3 × JBO 1, and JROBA 3 × JROBA 4) except for the parent JRO 204. Hybrid JROBA 3 × JRO 2407 had the highest fiber yield of 27 g/plant and the lowest fiber weight/plant was that of hybrid JRO 2407 × JROBA 4 (12.85 g). The average stick weight per plant of the hybrids was higher than that of the parents. It varied from 25.2 g/plant (JROBA 4 × JROM 1) to 59 g/plant (JROBA 3 × JROM 1). The green weight/plant of the hybrids was significantly higher than that of the parents, with an average of 267.84 g/plant. It varied from 171.34 g/plant (S 19 × JRO 2407) to 355.67 g/plant (JBO 1 × S 19).

**Table 2:** Analysis of variance of the diallel mating design for fiber yield and yield components.

	df	Plant height	Basal diameter	Fiber weight/plant	Stick weight/plant	Green weight/plant
Replication	2	2,069.67	2.36	7.19	73.45	1,154
Genotype	99	1,491.72***	8.47**	17.79**	139.01**	4,248**
Parents	9	5,284.03**	5.82*	20.34**	118.08**	1,060
Hybrids	89	1,042.40*	9.07**	17.65**	140.16**	4,612**
Parents vs hybrids	1	7361.31**	1.23	7.05	224.61**	573**
F1s	44	894.14	11.76**	21.35**	177.45**	4,434**
Reciprocals	44	1,198.28**	6.25**	13.93**	102.88**	4,642**
F1 vs reciprocals	1	707.29	15.09*	18.67**	139.32	11,114**
Error	198	699.54	3.30	2.25	28.13	1443.10

*P<0.05 (statistically significant at the 5% level), **P<0.01 (statistically significant at the 1% level).

**Table 3:** Mean performance of F_1_ progenies and parental lines for fiber yield and yield components.

Entry	Plant height (cm)	Basal diameter (mm)	Fiber weight/plant (g)	Stick weight/plant (g)	Green weight/plant (g)
JROBA 3	417.40	17.8	19.37	151.52	276.33
JROBA 3 × JRO 2407	454.00	24.3	27.00	168.43	344.67
JROBA 3 × JRO 8432	445.47	21.5	21.33	162.78	342.00
JROBA 3 × JRO 524	459.13	20.0	18.07	165.74	289.33
JROBA 3 × S 19	446.00	20.7	16.15	160.94	278.00
JROBA 3 × JROMU 1	422.00	18.2	16.20	152.12	247.67
JROBA 3 × JBO 1	458.87	22.1	20.95	167.32	347.67
JROBA 3 × JROBA 4	449.87	20.7	20.80	163.79	285.00
JROBA 3 × JROM1	416.00	17.8	20.65	151.48	269.00
JROBA 3 × JRO 204	431.67	19.0	17.50	156.07	242.33
JRO 2407 × JROBA 3	436.93	18.5	14.90	156.78	252.67
JRO 2407	455.33	20.1	13.13	162.86	284.67
JRO 2407 × JRO 8432	458.73	21.1	17.87	165.92	325.33
JRO 2407 × JRO 524	454.73	17.4	17.55	163.24	304.67
JRO 2407 × S 19	450.60	17.4	17.60	161.85	236.33
JRO 2407 × JROMU 1	430.60	17.4	15.15	154.40	204.00
JRO 2407 × JBO 1	450.93	18.3	20.45	163.23	233.33
JRO 2407 × JROBA 4	439.40	19.7	12.85	157.31	249.00
JRO 2407 × JROM 1	450.00	19.4	17.30	162.24	256.67
JRO 2407 × JRO 204	454.53	18.7	17.50	163.56	322.33
JRO 8432 × JROBA 3	427.00	19.8	19.80	155.52	253.17
JRO 8432 × JRO2407	454.33	20.5	20.13	164.99	285.67
JRO 8432	466.27	18.8	14.77	166.62	233.00
JRO 8432 × JRO524	452.67	18.7	15.27	162.23	260.93
JRO 8432 × S19	454.60	20.6	17.30	164.16	202.77
JRO 8432 × JROMU1	425.73	17.8	14.90	152.81	298.33
JRO 8432 × JBO1	446.60	19.0	18.20	161.27	292.70
JRO 8432 × JROBA 4	439.20	19.1	18.35	158.90	303.23
JRO 8432 × JROM 1	442.47	23.4	21.90	162.60	327.17
JRO8432 × JRO 204	428.60	19.8	18.90	155.77	254.50
JRO 524 × JROBA 3	436.00	19.9	18.45	158.10	286.37
JRO 524 × JRO 2407	430.07	17.4	17.30	154.93	233.67
JRO 524 × JRO 8432	455.13	18.7	18.55	164.14	263.43
JRO 524	428.47	19.7	14.36	154.17	243.67
JRO 524 × S 19	423.67	16.8	13.85	151.45	219.00
JRO524 × JROMU1	449.73	23.3	16.53	163.18	290.60
JRO 524 × JBO 1	459.40	22.8	20.05	167.41	320.77
JRO 524 × JROBA 4	451.20	22.4	19.05	164.22	337.00
JRO 524 × JROM 1	441.07	24.4	18.80	161.42	279.33
JRO 524 × JRO 204	425.67	20.3	17.20	154.39	246.83
S 19 × JROBA 3	454.53	17.4	15.50	162.49	255.70
S 19 × JRO 2407	431.20	19.1	13.15	154.48	171.33
S 19 × JRO 8432	456.47	20.6	15.50	164.18	287.90
S 19 × JRO 524	456.20	19.4	17.55	164.38	299.93
S 19	467.40	18.4	18.19	168.00	264.87
S 19 × JROMU 1	427.40	18.6	14.80	153.60	257.07
S 19 × JBO 1	428.40	17.0	13.25	152.87	222.73
S 19 × JROBA 4	451.53	18.2	15.65	161.81	287.80
S 19 × JROM 1	468.67	18.6	16.55	167.93	233.63
S 19 × JRO 204	430.60	19.4	15.45	155.14	261.33
JROMU 1 × JROBA 3	442.67	19.4	15.65	159.24	255.73
JROMU 1 × JRO 2407	449.07	19.5	14.97	161.19	274.30
JROMU 1 × JRO 8432	418.93	20.9	16.65	152.14	205.00
JROMU 1 × JRO 524	449.73	21.3	19.25	163.42	317.67
JROMU 1 × S 19	459.87	21.6	17.70	166.40	280.70
JROMU 1	428.53	18.5	14.66	153.89	258.93
JROMU 1 × JBO 1	407.00	17.7	16.00	146.90	242.07
JROMU 1 × JROBA 4	437.53	18.9	13.35	156.60	278.57
JROMU 1 × JROM 1	422.80	17.4	15.95	152.04	213.60
JROMU 1 × JRO 204	439.80	21.8	18.40	160.01	301.47
JBO 1 × JROBA 3	456.67	20.9	19.85	165.80	281.00
JBO 1 × JRO 2407	476.33	20.6	19.45	172.13	323.33
JBO 1 × JRO 8432	446.60	19.5	18.70	161.60	250.00
JBO 1 × JRO 524	478.40	20.9	15.60	171.64	326.00
JBO 1 × S 19	498.93	21.3	20.75	180.33	355.67
JBO 1 × JROMU 1	432.07	18.9	16.85	155.95	259.67
JBO 1	461.27	19.6	16.70	165.85	276.27
JBO 1 × JROBA 4	465.33	19.8	17.95	167.71	286.00
JBO 1 × JROM 1	462.40	20.4	18.90	167.23	281.67
JBO1 × JRO204	401.67	18.1	15.70	145.16	232.33
JROBA 4 × JROBA 3	448.93	18.7	14.20	160.61	283.90
JROBA 4 × JRO 2407	417.47	19.3	15.65	150.80	205.67
JROBA 4 × JRO 8432	421.20	17.5	15.20	151.31	267.83
JROBA 4 × JRO 524	434.13	18.2	16.95	156.43	243.27
JROBA 4 × S 19	425.00	16.6	16.55	152.70	203.33
JROBA 4 × JROMU 1	434.27	19.1	16.68	156.68	287.97
JROBA 4 × JBO1	465.33	21.1	15.30	167.24	260.33
JROBA 4	471.33	18.6	16.20	168.70	255.67
JROBA 4 × JROM1	401.33	18.8	14.03	144.73	280.37
JROBA 4 × JRO 204	449.20	20.8	16.75	162.25	288.50
JROM 1 × JROBA 3	448.13	21.3	20.35	163.27	257.00
JROM 1 × JRO 2407	472.87	19.7	19.75	170.79	287.67
JROM 1 × JRO 8432	435.40	18.3	16.75	156.80	213.00
JROM 1 × JRO 524	472.73	19.9	14.35	169.01	263.67
JROM 1 × S 19	445.13	19.2	20.50	161.62	288.67
JROM 1 × JROMU 1	453.33	21.6	20.70	165.21	277.00
JROM 1 × JBO 1	435.53	18.3	16.20	156.68	238.33
JROM 1 × JROBA 4	438.07	17.7	14.00	156.58	239.33
JROM 1	434.47	20.3	17.33	157.35	251.00
JROM 1 × JRO 204	406.53	20.6	16.05	147.73	284.67
JRO 204 × JROBA 3	428.53	18.3	17.70	154.86	263.33
JRO 204 × JRO 2407	425.20	16.7	16.95	152.95	255.47
JRO 204 × JRO 8432	429.93	18.7	17.15	155.25	212.67
JRO 204 × JRO 524	439.53	18.7	14.60	157.60	289.07
JRO 204 × S 19	433.73	16.5	13.15	154.47	171.40
JRO 204 × JROMU 1	408.47	20.1	15.35	147.97	235.67
JRO 204 × JBO 1	405.07	17.7	15.65	146.15	246.70
JRO 204 × JROBA 4	476.67	20.9	16.65	171.39	335.70
JRO 204 × JROM 1	417.20	17.0	13.75	149.32	239.00
JRO 204	458.80	21.2	21.70	167.25	292.53

Pearson's correlation revealed that all the fiber yield component traits were positively correlated with fiber yield (**Table 4**). Plant height has a positive correlation with other traits, and it ranged from 0.24 (P < 0.01) to 0.406 (P < 0.01). Stick weight showed a high positive correlation with fiber weight (0.731, P < 0.01) and green weight (0.609, P < 0.01). Additionally, green weight was significantly correlated with plant height, basal diameter, fiber weight, and stick weight.

**Table 4:** Correlation between studied traits.

	Plant height	Basal diameter	Fiber weight/plant	Stick weight/plant
Basal diameter	0.329**			
Fiber weight	0.249^*^	0.547^**^		
Stick weight	0.345^**^	0.458^**^	0.731^**^	
Green weight	0.406^**^	0.594^**^	0.517^**^	0.609^**^

*P<0.05 (statistically significant at the 5% level), **P<0.01 (statistically significant at the 1% level).

### Heterosis

BH, MH, and SH estimated values varied in magnitude (**Supplementary Tables 1**–**3**) and these variations were dependent on the hybrid cross combinations and the different traits studied. Average MH ranged from -3.29% (plant height) to 9.20% (stick weight). Many cross-combinations exhibited significant positive heterosis. The hybrid JROBA 4 × JROM 1 had the most significant negative heterosis for plant height (-19.80%), while JROM 1 × JRO 524 had the most significant positive heterosis (9.56%). Whereas, in the case of BH for plant height, it varied from -29.13% (JROBA 4 × JROM 1) to 8.8% (JROM 1 × JRO 524). SH for plant height ranged from -12.53% (JROBA 4 × JROM 1) to 8.75% (JBO 1 × S 19). Other crosses that showed significant positive heterosis were JBO 1 × JRO 524 (7.54%) and JBO 1 × S 19 (7.45%). Another important trait that has a direct impact on fiber yield is basal diameter. The highest significant negative MH for basal diameter was exhibited by the hybrid JRO 2407 × JRO 524 (-12.37%), whereas significant positive heterosis was recorded in JROBA 3 × JRO 2407 (28.18%). Cross combinations that exhibited significant positive heterosis were JROBA 3 × JRO 2407, JRO 8432 × JROM 1, JRO524 × JROMU 1, and JRO 524 × JBO 1. Basal diameter combined with plant height is a good indicator of high fiber yield. Alone, high plant height does not significantly affect fiber yield in jute. However, a longer length of mid fiber is of better quality. Fiber weight is the most economical trait for jute. The jute yield plateau can only be broken if fiber weight/plant increases significantly. Thus, positive heterosis is desirable for this trait. Many crosses showed positive heterosis, depicting that the hybrids had favorable gene combinations for most of the loci. MH for fiber weight varied from -34.08% (JRO 204 × S 19) to 66.15% (JROBA 3 × JRO 2407). Out of 90 hybrids, 31, 20, and 2 hybrids displayed significant positive MH, BH, and SH, respectively. Whereas, in the case of BH for fiber weight, it varied from -39.40% (JRO 204 × S 19) to 39.41% (JROBA 3 × JRO 2407). SH for fiber weight ranged from -40.79% (JRO 2407 × JROBA 4) to 24.42% (JROBA 3 × JRO 2407). The highest significant negative MH for stick weight was exhibited by the hybrid JRO 204 × S 19 (-30.74%), whereas highest significant positive heterosis was recorded in JRO 2407 × JRO 524 (59.85%). Cross combinations that exhibited very high (greater than 30%) significant positive MH were JROBA 3 × JRO 2407, JRO 2407 × JBO 1, JRO 8432 × JRO 2407, JRO 8432 × JROM 1, JROMU 1 × JRO 524, and JBO 1 × JRO 2407. BH for stick weight varied from -36.00% (JRO 204 × JROM 1) to -57.97% (JRO 2407 × JRO 524), whereas SH for stick weight ranged from -46.95% (JROBA 4 × JROM 1 and JROMU 1 × JROBA 4) to 24.21% (JROBA 3 × JROM 1). In the case of stick weight, 26 and 6 hybrids displayed significant positive BH and SH, respectively. Primarily, jute is grown for fiber but in Kenya, Bangladesh, and India it is also grown for leafy vegetables. A high yield of leafy vegetables corresponds to a genotype's high biomass/green weight. In this study, MH for green weight varied from -38.50% (JRO 204 × S 19) to 35.19% (JRO 8432 × JROM 1), while MH and SH ranged from -41.41% (JRO 204 × S 19) to 30.35% (JRO 8432 × JROM 1) and -41.43% (S 19 × JRO 2407) to 18.85% (JROBA 3 × JBO 1). Finally, 28, 15, and 10 hybrids displayed significant positive MH, BH, and SH respectively.

### Combining ability

The ANOVA for the diallel mating design is presented in **Table 2**. The variance attributed to parents was statistically significant (P < 0.01) for all traits except green weight. Variances due to genotypes, hybrids, and reciprocal crosses were significant for all the traits studied. Variance due to F_1s_ and F_1s_ vs. reciprocals was significant for all the traits except plant height. The variance attributed to the parents vs. hybrids was statistically significant for all traits except basal diameter.

No parents were found to have positive GCA effects for all traits concurrently, except JBO 1 as shown in **Table 5**. Six parents (JROBA 4 > JBO 1 > S 19 > JRO 2407 > JRO 524 > JRO 8432 > JRO M1 > JRO MU 1 > JRO 204 > JROBA 3) had positive GCA effects for plant height. JRO 524 only exhibited significant positive GCA effects for basal diameter, and in total, 6 parents had positive GCA effects for this trait (JRO 524 > JRO M 1 > JROBA 3 > JBO 1 > JRO 8432 > JROMU 1). Four out of 10 parents, namely JROBA 3, JBO 1, JRO 8432, and JROM 1, showed highly significant positive GCA effects for fiber weight. JROBA 3 exhibited positive and significant results for all the fiber yield-related traits, namely fiber weight, stick weight, and green weight. JROMU 1, JROBA 4, and JROM 1 showed significant negative GCA effects for stick weight. Three parents, viz., JROBA 3, JRO 524, and JBO 1, showed highly significant positive GCA effects for stick weight.

**Table 5:** Estimation of GCA effects of parents for fiber yield and yield components.

Parent	Plant height (cm)	Basal diameter (mm)	Fiber weight/plant (g)	Stick weight/plant (g)	Green weight/plant (g)
JROBA 3	-3.7059	0.2226	1.5817**	3.5592**	11.5190*
JRO 2407	3.8174	-0.2162	-0.0184	1.1617	-1.0693
JRO 8432	0.0141	0.1779	0.4920**	0.9275	-2.2593
JRO 524	2.7407	0.5133*	-0.2229	1.1283	10.1023*
S 19	5.3007	-0.7015**	-0.7406**	0.1575	-15.6893**
JROMU 1	-10.162**	0.0351	-0.8871**	-3.0233**	-5.5943
JBO 1	6.3374	0.1964	0.5526**	0.1725	9.8007*
JROBA 4	10.3507 **	-0.251	-0.9895**	-2.0975**	3.8657
JROM 1	-3.6360	0.2333	0.4498**	-1.6458*	-6.2527
JRO 204	-11.0560 **	-0.2098	-0.2175	-0.3400	-4.4226

*P<0.05 (statistically significant at the 5% level), **P<0.01 (statistically significant at the 1% level).

As observed for GCA, none of the hybrid combinations had simultaneous positive SCA (specific combining ability) effects for all the traits studied (**Table 6**). Hybrids such as JBO 1 × S 19 and JBO 1 × JRO 204 exhibited simultaneous negative SCA effects for all the traits. Among these traits, stick weight (60%) exhibited the highest proportion of crossings with favorable SCA effects, while basal diameter (51%) had the lowest proportion. Of the crosses, 58% had positive effects on fiber weight, followed by plant height (54%) and green weight (53%). For plant height, the SCA effect ranged from -35.48 (JBO 1 × JRO 204) to 17.68 (JRO 2407 × JROM 1), whereas for basal diameter, it ranged from -2.35 (JRO 2407 × JRO 524) to 2.89 (JRO 2407 × JROBA 3), -3.75 (JBO 1 × S 19) to 3.12 (JRO 2407 × JROBA 3), -12.86 (JBO 1 × S 19) to 12.53 (JROM 1 × JROBA 3), -66.46 (JBO 1 × S 19) to 57.08 (JROM 1 × JRO 8432), fiber weight, stick weight, and green weight, respectively. Crosses such as JROM 1 × JROMU 1, JBO 1 × JRO 204, JBO 1 × S 19, S 19 × JRO 204, and JRO 2407 × JROBA 4 showed negative SCA for all the traits studied. However, the best crosses in terms of SCA effects were JRO 2407 × JROBA 3, JRO 2407 × JRO 8432, JRO 524 × JROMU 1, JROBA 4 × JRO 524, JROBA 4 × JRO 204, and JROM 1 × JRO 8432.

**Table 6:** Estimation of relative SCA effects of hybrids for fiber yield and yield components.

Entry	Plant height	Basal diameter	Fiber weight/plant	Stick weight/plant	Green weight/plant
JROBA 3 × JRO 2407	1.7893	1.9146**	2.2791 **	3.1033	20.3760
JROBA 3 × JRO 8432	-3.6407	0.7652	1.3854 **	6.9375 **	20.4827
JROBA 3 × JRO 524	4.9659	-0.2855	-0.2080	-2.9967	-1.6123
JROBA 3 × S 19	5.1060	0.0366	-2.1237 **	-5.2258 **	3.1793
JROBA 3 × JROMU 1	2.6359	-0.9503	-1.8771 **	-1.6700	-22.0657
JROBA 3 × JBO 1	11.5693	1.6124	1.1581 *	1.7758	25.1727
JROBA 3 × JROBA 4	-0.8107	0.2325	-0.1997	-3.4125	1.2243
JROBA 3 × JROM1	-4.1574	-0.3778	1.3610 **	5.2608 **	-10.1073
JROBA 3 × JRO 204	1.2959	-0.8051	-0.8718	-1.0283	-22.1040
JRO 2407 × JROBA 3	8.5333	2.8913 **	6.0500 **	5.8833 **	46.0000 **
JRO 2407 × JRO 8432	9.1359	1.3753 *	1.4189 **	5.6350 **	40.9876 **
JRO 2407 × JRO 524	-7.7241	-2.3501 **	0.5587	4.0342 *	-7.7074
JRO 2407 × S 19	-11.7840	-0.3450	-0.9736	-3.8950 *	-47.2491 **
JRO 2407 × JROMU 1	2.6126	-0.8139	-1.1437 *	-2.3392	-22.0274
JRO 2407 × JBO 1	9.9126	-0.0075	2.3082 **	7.5900 **	1.7610
JRO 2407 × JROBA 4	-29.3007**	0.4732	-1.8496 **	-4.1067 *	-43.3040 **
JRO 2407 × JROM 1	17.6860	0.0722	0.9861	2.7417	11.6476
JRO 2407 × JRO 204	3.5393	-1.3903 *	0.3533	-1.5808	26.5510
JRO 8432 × JROBA 3	9.2333	0.8867	0.7667	5.5500 *	44.4167 **
JRO 8432 × JRO2407	2.20	0.3247	-1.1333 *	0.5167	19.8333
JRO 8432 × JRO524	7.5793	-1.4301 *	-0.4683	0.1517	-13.5007
JRO 8432 × S19	6.6527	1.6206 *	-0.4590	0.2225	-4.5590
JRO 8432 × JROMU1	-11.0841	-0.3750	-0.9374	-1.5467	-8.3207
JRO 8432 × JBO1	-3.3174	-0.6166	0.2978	-2.2092	-4.0323
JRO 8432 × JROBA 4	-23.7307*	-1.0719	0.1650	2.5275	16.0860
JRO 8432 × JROM 1	-1.0107	0.9562	1.2757 *	-3.7075	10.7543
JRO8432 × JRO 204	-3.2574	-0.2124	0.6429	-0.5300	-27.5757 *
JRO 524 × JROBA 3	11.5667	0.0767	-0.1917	2.6500	1.4833
JRO 524 × JRO 2407	12.33	0.0087	0.1250	3.6167	35.5000 *
JRO 524 × JRO 8432	-1.2333	0.2122	-1.6417 **	2.345	-1.250
JRO 524 × S 19	-11.674	-1.1901	-0.4441	-3.3283	-2.7873
JRO524 × JROMU1	13.5893	2.2373 **	1.8941 **	4.6025 *	31.7843 *
JRO 524 × JBO 1	16.256	1.6654 *	0.3877	2.5067	35.6393 *
JRO 524 × JROBA 4	-13.9907	0.5511	2.1049 **	5.4517 **	8.3243
JRO 524 × JROM 1	14.2293	1.9325 **	-0.7594	2.9167	-0.1907
JRO 524 × JRO 204	-2.6507	-0.3011	-0.7672	-2.9558	-5.5707
S 19 × JROBA 3	-4.2667	1.6153 *	0.3250	0.2833	11.1500
S 19 × JRO 2407	9.70	-0.8630	2.2250 **	-0.2833	32.5000 *
S 19 × JRO 8432	-0.9333	0.3136	0.9000	0.2500	-42.5667 **
S 19 × JRO 524	16.2667	-1.2680	-1.8500 **	-7.1500 **	-40.4667 **
S 19 × JROMU 1	4.9293	1.2921	0.7701	3.4233	22.3260
S 19 × JBO 1	8.4627	0.1522	0.0804	3.7108	27.2477
S 19 × JROBA 4	-20.9507*	-1.1391	0.7225	1.9225	-10.4507
S 19 × JROM 1	11.6693	-0.1168	1.7083 **	4.4375 *	15.2510
S 19 × JRO 204	-5.6440	-0.6327	-1.8495 **	-5.9933 **	-31.3624 *
JROMU 1 × JROBA 3	-10.3333	-0.6210	0.2750	1.7083	-4.0333
JROMU 1 × JRO 2407	-9.23	-1.0493	0.0917	-5.6583 **	-35.1500 *
JROMU 1 × JRO 8432	3.4000	-1.5257 *	-0.8750	0.3500	46.6667 **
JROMU 1 × JRO 524	5.8923	0.9933	-1.3583 *	-4.5000 *	-13.5333
JROMU 1 × S 19	-16.2333	-1.5120 *	-1.4500 *	-4.8500 *	-11.8167
JROMU 1 × JBO 1	-20.2073*	-1.4015 *	-0.3481	-4.1667 *	-21.1807
JROMU 1 × JROBA 4	-7.8540	-0.2661	-0.2142	-2.8467	17.1543
JROMU 1 × JROM 1	8.2993	-0.2737	1.6548 **	-0.6233	-10.6940
JROMU 1 × JRO 204	1.7860	1.6410 *	0.8720	4.7458 *	10.7426
JBO 1 × JROBA 3	1.10	0.6250	0.5500	4.0333	33.3333 *
JBO 1 × JRO 2407	-12.70	-1.1417	0.5000	-4.8167 *	-45.0000 **
JBO 1 × JRO 8432	2.36	-0.2453	-0.2500	2.4833	21.3500
JBO 1 × JRO 524	-9.5000	0.9320	2.2250 **	5.6000 **	-2.6167
JBO 1 × S 19	-35.2667 **	-2.1680 **	-3.7500 **	-12.8667 **	-66.4667 **
JBO 1 × JROMU 1	-12.5333	-0.6117	-0.4250	-2.8250	-8.8000
JBO 1 × JROBA 4	5.0793	1.0370	-0.0456	-0.6592	-8.3407
JBO 1 × JROM 1	2.6993	-0.5730	-0.5599	1.4308	-11.3890
JBO1 × JRO204	-35.4807**	-1.5649 *	-1.7677 **	-5.0083 *	-33.7024 *
JROBA 4 × JROBA 3	0.4667	1.0063	3.3000 **	7.2917 **	0.5500
JROBA 4 × JRO 2407	10.9667	0.1990	-1.4000 *	2.7500	21.6667
JROBA 4 × JRO 8432	9.00	0.8033	1.5750 **	6.5000 **	17.7000
JROBA 4 × JRO 524	8.5333	2.1077 **	1.0500	7.2750 **	46.8667 **
JROBA 4 × S 19	13.2667	0.8440	-0.4500	0.2750	42.2333 **
JROBA 4 × JROMU 1	1.6333	-0.0950	-1.6667 **	-6.1250 **	-4.7000
JROBA 4 × JBO1	4.133	-0.6300	1.3250 *	3.0417	12.8333
JROBA 4 × JROM1	-30.5807**	-1.2156	-2.5511 **	-5.7825 **	-5.6040
JROBA 4 × JRO 204	20.0726*	1.8025 **	0.7994	4.6700 *	44.8160 **
JROM 1 × JROBA 3	-16.0667	-1.7657 *	0.1500	12.5333 **	6.0000
JROM 1 × JRO 2407	11.4333	-0.1643	-1.2250 *	-4.1167	-15.5000
JROM 1 × JRO 8432	3.5333	2.5930 **	2.5750 **	6.2333 **	57.0833 **
JROM 1 × JRO 524	-15.8333	2.2260 **	2.2250 **	1.6583	7.8333
JROM 1 × S 19	11.7667	-0.3213	-1.9750 **	-6.6417 **	-27.5167
JROM 1 × JROMU 1	-15.2667	-2.1070 **	-2.3750 **	-8.6500 **	-31.7000 *
JROM 1 × JBO 1	13.4333	1.0490	1.3500 *	2.1167	21.6667
JROM 1 × JROBA 4	-18.3667	0.5790	0.0167	-4.5667 *	20.5167
JROM 1 × JRO 204	-17.0074	-0.7051	-2.4399 **	-1.2067	4.6677
JRO 204 × JROBA 3	1.5667	0.3513	-0.1000	3.5833	-10.5000
JRO 204 × JRO 2407	14.6667	0.9840	0.2750	4.3333 *	33.4333 *
JRO 204 × JRO 8432	-0.6667	0.5740	0.8750	2.7500	20.9167
JRO 204 × JRO 524	-6.9333	0.8247	1.3000 *	-0.7250	-21.1167
JRO 204 × S 19	-1.5667	1.4143	1.1500 *	1.3167	44.9667 **
JRO 204 × JROMU 1	15.6667	0.8640	1.5250 **	2.3250	32.9000 *
JRO 204 × JBO 1	-1.7000	0.1907	0.0250	2.2167	-7.1833
JRO 204 × JROBA 4	-13.7333	-0.0253	0.0500	1.2750	-23.6
JRO 204 × JROM 1	-5.3333	1.7907 *	1.1500 *	5.7000 **	22.83

*P<0.05 (statistically significant at the 5% level), **P<0.01 (statistically significant at the 1% level).

### Estimation of genetic parameters

The variance attributed to specific combining ability (σ^2^ SCA) was greater than general combining ability (σ^2^ GCA) for all the traits (**Table 7**). The dominance genetic variance had a greater magnitude than the additive genetic variance for all the characters studied. The results were substantiated by the GCA variance ratio (σ^2^ GCA/σ^2^ SCA) and the degree of dominance (σ^2^ D/σ^2^ A) as traits with higher GCA have a GCA variance ratio greater than 1. H^2^ ranged from 66.65% (plant height) to 37.20% (green weight). However, all the traits showed very low values of h^2^, ranging from 3.38 (basal diameter) to 12.47% (fiber weight).

**Table 7:** Estimation of genetic variances and heritability for fiber yield and yield components.

Parameters	Plant height	Basal diameter	Fiber weight/plant	Stick weight/plant	Green weight/plant
σ^2^ GCA	38.3993	0.0655	0.5963	3.1497	52.4148
σ^2^ SCA	408.4881	1.7344	4.1698	28.5321	561.3181
σ^2^ A	19.20	0.03275	0.29815	1.57485	26.2074
σ^2^ D	408.4881	1.7344	4.1698	28.5321	561.3181
σ^2^_GCA/_σ^2^_SCA_	0.0940	0.0378	0.1430	0.1104	0.0934
σ^2^ D/σ^2^ A	21.27	52.95	13.98	18.11	21.41
(σ^2^ D/σ^2^ A)^1/2^	4.61	7.27	3.74	4.25	4.62
H^2^ (%)	66.65	48.27	49.12	39.39	37.20
h^2^ (%)	10.53	3.38	12.47	7.15	5.85

### Genotyping

**Figure 1:**
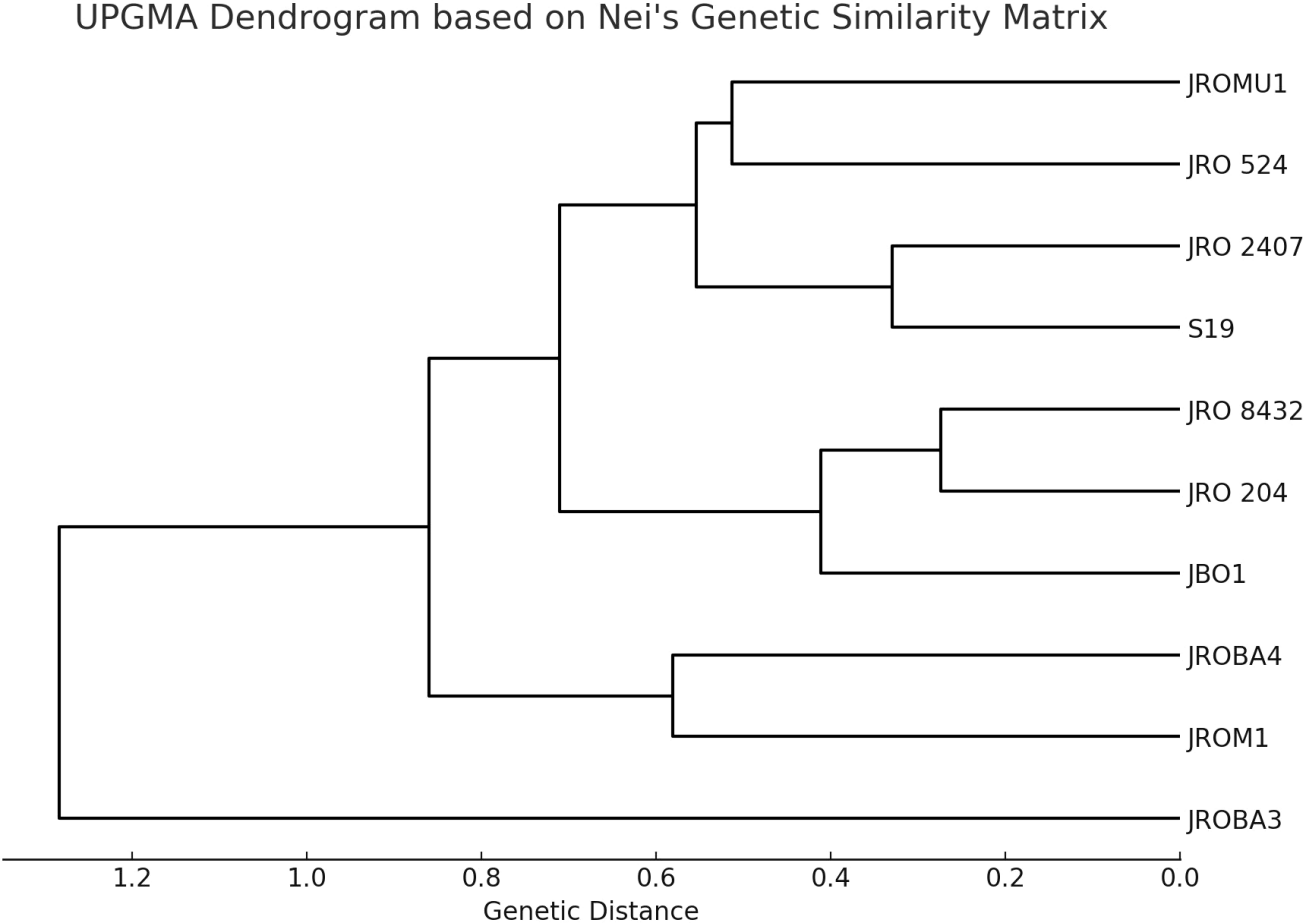
UPGMA dendrogram based on Nei's genetic similarity matrix.

Initially, 70 ILP markers were amplified, of which only 12 (17%) were polymorphic among the 10 genotypes selected for analysis. The genetic diversity of the genotypes was analyzed to confirm significant divergence between the parents for GCA and SCA analysis. However, the research's basic objective was to explore hybridization in the jute breeding program. Nei's genetic similarity index (Nei, 1972) was calculated to decipher the genetic relationships. The values in the matrix ranged from 0.182 to 1.000, with 1.000 showing complete genetic identity, and lower values showing higher genetic divergence (**Table 8**). The genotypes JRO 204 and JRO 8432 showed a genetic similarity of 0.926, indicating their closeness. The same was also found for JRO 204, which had high genetic similarity with genotype JRO 524 at a level of 0.857, which further indicates its closeness to genetically related varieties. Genotypes JROM1 and JROBA 3 had the lowest genetic similarity index of 0.183, implying a wide gap in genetic similarity. This analysis also revealed a moderate divergence of 0.772 between JROM 1 and JROBA 4, and 0.617 between S19 and JRO 8432.The pattern of clustering is shown very clearly in **Figure 1**, which is a dendrogram made using Nei's genetic similarity index and the genotypes of *C.olitorius*. The dendrogram clearly displays that two major clusters exist. The genotypes of the first group are JRO 204, JRO 524, and JRO 8432. The genotypes reasonably cluster together with substantial genetic similarity, as reflected by the similarity matrix also. The second group included JROM 1, JROMU 1, JROBA 3, JROBA 4, and JRO 2407 genotypes. However, the similarity index supports the conclusion that JROM 1 and JROBA 3 exhibit significant differences in this group.

**Table 8:** Nei's genetic similarity matrix.

	JRO 204	JRO 524	JRO 8432	JBO1	JROM1	S19	JRO 2407	JROMU1	JROBA 3	JROBA4
JRO 204	1	0.857143	0.92582	0.857143	0.771517	0.714286	0.668153	0.629941	0.507093	0.571429
JRO 524	0.857143	1	0.771517	0.714286	0.617213	0.714286	0.801784	0.755929	0.338062	0.714286
JRO 8432	0.92582	0.771517	1	0.771517	0.666667	0.617213	0.57735	0.544331	0.547723	0.46291
JBO1	0.857143	0.714286	0.771517	1	0.617213	0.571429	0.534522	0.629941	0.676123	0.428571
JROM1	0.771517	0.617213	0.666667	0.617213	1	0.46291	0.433013	0.680414	0.182574	0.771517
S19	0.714286	0.714286	0.617213	0.571429	0.46291	1	0.801784	0.629941	0.507093	0.571429
JRO 2407	0.668153	0.801784	0.57735	0.534522	0.433013	0.801784	1	0.707107	0.474342	0.668153
JROMU1	0.629941	0.755929	0.544331	0.629941	0.680414	0.629941	0.707107	1	0.447214	0.755929
JROBA 3	0.507093	0.338062	0.547723	0.676123	0.182574	0.507093	0.474342	0.447214	1	0
JROBA4	0.571429	0.714286	0.46291	0.428571	0.771517	0.571429	0.668153	0.755929	0	1

## Discussion

Significant differences among the 10 jute parents revealed adequate genetic diversity in plant height, basal diameter, fiber weight, stick weight, and green weight, indicating a strong potential for improving these traits through breeding. Significant genetic diversity among jute germplasm has been observed, with stick weight and fiber weight accounting for over 75% of the total variability (Jatothu et al., 2018). However, germplasm evaluation for fiber yield-related traits reported limited genetic variability among tossa jute genotypes (Ghosh et al., 2017 and Mangal et al., 2023). Zhang et al. (2015) asserted that the genetic variation of jute accessions was large and genetic similarity coefficients varied from 0.520 to 0.910. In the current study, the significant differences observed among parents may be due to differences in pedigree. Therefore, hybrids developed from these parents would broaden the genetic base for the jute improvement program and the chances of recovery of good recombinants would be high.

Fiber yield, a multifaceted quantitative characteristic, is regulated and influenced by various yield components, including green and stick weights. The outcomes obtained align with prior findings (Hassan et al., 2024). The findings indicate that fiber weight exhibited significant associations with stick weight, green weight, and plant height (Sharma et al., 2016). Our trial revealed that high-yielding entries exhibited longer height, greater green weight, and stick weight than lower-yielding entries. In general, the hybrids demonstrated superior performance in fiber yield and yield-related characteristics compared to the parental lines, clearly displaying hybrid vigor. The estimated heterosis values for SH, MPH, and BPH showed different degrees, consistent with previously reported findings (**Supplementary Table 1**). MPH had the highest average values for the traits studied, followed by SH and BPH, as observed in various studies (Palve et al., 2003). SH is the most pertinent heterosis value in terms of realism, as it determines the possible outyielding ability of hybrids over the locally accepted commercial cultivar. An acceptable figure for hybrids in natural fiber crops is a 10% to 15% yield advantage over the best variety. In our investigation, some crosses exhibited standard heterosis of 15% for fiber yield. JROBA 3 × JRO 2407 has a significant advantage of 24.42% over the high-yielding national check variety JRO 204. Whereas, in the use of jute for vegetable purposes, hybrid JROBA 3 × JBO 1 showed a yield advantage of 18.85% over JRO 204. Furthermore, considering that the comparison was conducted using the best commercial cultivar, our findings unequivocally demonstrate the viability of these crosses for commercial purposes and breaking the yield plateau in jute.

Choosing appropriate parents and understanding the genetic mechanisms behind desired features are crucial methods for enhancing genetic quality and creating new hybrids in jute. Estimated GCA effects aid in identifying parents possessing favorable genetic potential for producing progeny with desirable features. Out of the parents used in the hybridization program in our study, JROBA 3 exhibited significant potential with the highest GCA for fiber weight and performed well for yield component traits. Additionally, JBO 1 showed positive GCA effects across all traits. This outcome was superior to prior studies where no individual parent showed a significant GCA effect for all variables under investigation (Sawarkar et al., 2023). The hybrid JROBA 3 × JRO 2407 was the highest yielder among the 90 hybrids evaluated. Crosses involving JROBA 3 as parents yielded an average fiber weight of 18.85 g/plant. Additionally, the study noted that JROBA 3 effectively combined most of the yield component features, except for plant height. Therefore, JROBA 3 is a good general combiner for fiber weight.

Upon analyzing the crosses, it was found that no one combination yielded positive SCA values for all of the assessed traits. This finding is consistent with the results of previous research on jute and other fiber crops (Khatun et al., 2010; Sharma et al., 2016; Sawarkar et al., 2023; Hassan et al., 2024). No significant effects of SCA were observed in any of the crosses for all the traits simultaneously. This suggests that the values for these characteristics fall within the average range of the parents. As evident from previous studies, proficient general combiners produced the most superior crosses in relation to SCA. The best three crosses for fiber yield in terms of SCA effects are JRO 2407 × JROBA 3, JROBA 4 × JROBA 3, and JROM 1 × JRO 8432. JROBA 3 is a common parent among these crosses, providing favorable gene combinations with other parents. In some cases, it is interesting that good-by-good general combiners did not make the best SCA crosses. Such is the case for JRO 524 × JROMU 1, JRO 524 × JROBA 4, and S 19 × JRO 2407, all of which had positive SCA for fiber weight from parents with negative GCA, with one explanation being good complementary gene actions. JBO 1 × JRO 8432 and JBO 1 × JROM 1, however, had negative SCA effects despite having parents with good GCA for fiber weight, likely due to unfavorable combinations of genes or alleles from the parents. JROM 1 × JRO 8432 was the best specific combiner, and this hybrid may be used to recover transgressive segregants for breeding for high biomass, particularly for vegetable purposes. Both the parents of this cross have positive GCA effects, indicating a favorable combination of genes in the hybrid (Gramaje et al., 2020). Crosses with high SCA values are best for the exploitation of heterosis. While choosing hybrids with high SCA effects may not directly improve self-pollinated crops such as jute, which typically exhibit lower genetic variation, it is possible to identify transgressive segregants from the top-performing hybrids and fix them in future generations.

The σ^2^ D value was greater than σ^2^ A and σ^2^ SCA was greater than σ^2^ GCA for all the traits. This depicts the predominance of non-additive gene action. Moreover, the degree of dominance was greater than 1 for all the traits. The heritability provided further support for the findings since the h^2^ values were less than 10% for all traits except fiber weight and noticeably lower than the H^2^ values. Additionally, this supports the assumption of non-additive gene action. Significantly, basal diameter exhibited low levels of heredity, as shown by both h^2^ and H^2^. This outcome was anticipated due to the little variability seen in the collected data for the trait. Sharma et al. (2016) and Roy et al. (2020) have also shown the substantial influence of non-additive gene activity on the yield component traits, highlighting their significance in the development of hybrid jute. Selection for desirable genotypes based on phenotypic performance may out to be futile for these traits. Selection may be conducted by family or progeny testing, and it is recommended that this process be carried out in subsequent generations. We observed significant GCA and SCA variance for all the traits, signifying the importance of both additive and non-additive gene action. Khan et al. (2009); Sharma et al. (2016), and Abdel-Aty et al. (2023) also observed similar trends in fiber crops.

The genetic similarity matrix and UPGMA dendrogram showed that *C.olitorius* genotypes possess a high degree of genetic diversity based on intron-length polymorphic markers. The first cluster consisted of JRO 204, JRO 524, and JRO 8432 and indicated a close evolutionary relationship amongst the constituents of this cluster, likely because of common genetic resources or intentional breeding. The second group comprised JROM 1, JROMU 1, JROBA 3, JROBA 4, and JRO 2407, but JROM 1 and JROBA 3 had shown the highest genetic distance in the group. The mean value of the genetic similarity index for the populations was reported to be approximately 0.623. This means mid-level genetic similarity in the population included in the sample. This is in line with Huang et al. (2009), who postulated that self-pollinated crops such as jute have less variation within a population than cross-pollinated crops. In general, it has been assumed that jute crops are not susceptible to interspecific cross-hybridization and, therefore, have relatively less genetic diversity (Akter et al., 2008; Mir et al., 2008). Perhaps the low gene diversity for jute species might be due to high degrees of domestication and selection processes.

High-yielding and climate-resilient varieties capable of producing more than 35 quintals/hectare under a wide range of agro-climatic conditions should be the focus area of future research on jute breeding. Introducing new germplasm into these programs and promoting farmer adoption through participatory breeding will increase genetic diversity and ensure the successful implementation and sustainability of these developments in jute cultivation.

### Future prospects

Efforts should focus on broadening the genetic base through additional hybridization and advanced molecular techniques, such as genome-wide association studies (GWASs), to identify more genetic factors contributing to improved jute traits. Also, research in the area of fiber recovery optimization and sustainability aspects can further reduce the bottleneck in jute productivity.

## Conclusion

This study demonstrates the potential of hybridization and combining ability analysis to break the jute yield plateau, meeting the growing global demand for sustainable natural fibers. This research identifies superior parental lines and cross combinations that express positive general and specific combining abilities through diallel mating, which are very important for improving traits such as the yield of fiber and biomass accumulation. Our observations indicate that hybrid vigor or heterosis is the largest factor in some hybrids out-yielding their parents in fiber yield and other agronomic traits. The cross JROBA 3 × JRO 2407 exhibited the highest SCA, with a notable yield advantage of 24.42% over the national check variety, JRO 204. Therefore, this implies that these hybrids may make a significant contribution to jute fiber production. The study also underscores the importance of genetic diversity and the role of non-additive gene action, suggesting that heterosis should be exploited in breeding programs.

## Data Availability

The original contributions presented in the study are included in the article/**Supplementary Material**. Further inquiries can be directed to the corresponding authors.

## References

[B1] Abdel-Aty M. S. Sorour F. A. Yehia W. M. B. Kotb H. M. K. Abdelghany A. M. Lamlom S. F.. (2023). Estimating the combining ability and genetic parameters for growth habit, yield, and fiber quality traits in some Egyptian cotton crosses. BMC Plant Biol. 23. doi: 10.1186/s12870-023-04131-z PMC9979479 36859186

[B2] Adeyemo O. A. Ayodele O. O. Ajisafe M. O. Okinedo U. E. Adeoye D. O. Afanou A. B.. (2021). Evaluation of dark jute SSR markers and morphological traits in genetic diversity assessment of jute mallow (Corchorus olitorius L.) cultivars. South Afr. J. Bot. 137, 290–297. doi: 10.1016/J.SAJB.2020.10.027

[B3] Akter J. Sajib A. A. Ashraf N. Haque S. Khan H. (2008). Microsatellite markers for determining genetic identities and genetic diversity among jute cultivars. Aust. J. Crop Sci. South. Cross Journals© 1, 97–107.

[B4] Alimuzzaman S. Arin M. R. A. Mamun M. A. A. Rahman A. N. M. M. Islam M. R. (2024). A novel approach of manufacturing sustainable seamless jute bags and evaluation of its properties: A comparative study with commercial bags. J. Natural Fibers 21. doi: 10.1080/15440478.2023.2299548

[B5] Chourasia K. N. Patil V. U. Vanishree G. Kumar R. V. Thribhuvan R. Meena J. K.. (2023). Morphological and molecular characterization of Indian potato (Solatium tuberosum L.) cultivars. Cytol Genet. 57, 246–257. doi: 10.3103/S0095452723030039/METRICS

[B6] Commission for Agricultural Costs & Prices (2021). Price policy for jute: 2022-23 season (New Delhi: Ministry of Agriculture & Farmers Welfare, Government of India), 1–111.

[B7] Doyle J. J. Doyle J. L. (1987). A rapid DNA isolation procedure for small quantities of fresh leaf tissue. Phytochem. Bull. 19, 11–15.

[B8] Ghosh S. Meena K. Sinha M. K. Karmakar P. G. (2017). Genetic diversity in corchorus olitorius genotypes using jute SSRs. Proc. Natl. Acad. Sci. India Section B - Biol. Sci. 87, 917–926. doi: 10.1007/s40011-015-0652-4

[B9] Gramaje L. V. Caguiat J. D. Enriquez J. O. S. dela Cruz Q. D. Millas R. A. Carampatana J. E.. (2020). Heterosis and combining ability analysis in CMS hybrid rice. Euphytica 216. doi: 10.1007/s10681-019-2542-y

[B10] Griffing B. (1956). Concept of general and specific combining ability in relation to diallel crossing systems. Aust. J. Biol. Sci. 9, 463–493. doi: 10.1071/BI9560463

[B11] Hassan A. Ashraf J. Wahid S. Alyas K. Nisar S. Kanwal S.. (2024). Estimation of heterosis and combining ability for yield and fiber related traits in gossypium hirsutum L. Sarhad J. Agric. 40, 325–334. doi: 10.17582/journal.sja/2024/40.2.325.334

[B12] Huang Y. Zhang C. Q. Li D. Z. (2009). Low genetic diversity and high genetic differentiation in the critically endangered Omphalogramma souliei (Primulaceae): implications for its conservation. J. Syst. Evol. 47, 103–109. doi: 10.1111/J.1759-6831.2009.00008.X

[B13] Jatothu J. L. Kumar A. A. Choudhary S. B. Sharma H. K. Maruthi R. T. Kar C. S.. (2018). Genetic diversity analysis in tossa jute (Corchorus olitorius L.) germplasm lines. J. Agri. Nat. Sci. 10, 1–9. doi: 10.31018/jans.v10i1.1566

[B14] Jinks J. L. (1953). The analysis of diallel crosses. Maize Genet. Coop News Letter 27, 48–54. doi: 10.1038/hdy.1956.1

[B15] Jo B. S. Choi S. S. (2015). Introns: The functional benefits of introns in genomes. Genomics Inf. 13, 112–118. doi: 10.5808/GI.2015.13.4.112 PMC4742320 26865841

[B16] Khan N. U. Hassan G. Kumbhar M. B. Marwat K. B. Khan M. A. Parveen A.. (2009). Combining ability analysis to identify suitable parents for heterosis in seed cotton yield, its components and lint % in upland cotton. Ind. Crops Prod 29, 108–115. doi: 10.1016/j.indcrop.2008.04.009

[B17] Khatun R. Sarker R. H. Sobhan M. A. (2010). Combining ability for yield and yield contributing characters of white jute (Corchorus Capsularis L.). Bangladesh J. Bot. 39, 79–85. doi: 10.3329/bjb.v39i1.5530

[B18] Kostygov A. Y. Skýpalová K. Kraeva N. Yurchenko V. Gertman I. Tikhonenkov D.. (2024). Comprehensive analysis of the Kinetoplastea intron landscape reveals a novel intron-containing gene and the first exclusively trans-splicing eukaryote. BMC Biol. 22, 281. doi: 10.1186/s12915-024-02080-z 39627879 PMC11613528

[B19] Li D. Xia Z. Deng Z. Liu J. Zhang X. Chen W.. (2013). Development, characterization, genetic diversity, and cross-species/genera transferability of ILP markers in rubber tree (*Hevea brasiliensis*). Genes Genomics 35, 719–731. doi: 10.1007/s13258-013-0122-4

[B20] Liang G. H. (1972). Heterosis, inbreeding depression, and heritability estimates in a systematic series of grain sorghum genotypes1. Crop Sci. 12, 409–411. doi: 10.31018/jans.v8i1.771

[B21] Mangal V. Bhandari H. Meena J. K. Kumar A. A. Thribhuvan R. Chourasia K. N.. (2023). Genetic diversity analysis for seed yield attributing traits in white jute (Corchorus capsularis L.). Indian J. Plant Genet. Resour. 36, 31–36. doi: 10.5958/0976-1926.2023.00036.1.04

[B22] Manivannan N. (2014). TNAUSTAT – Statistical package. Available online at: https://sites.google.com/site/tnaustat (Accessed March 27, 2024).

[B23] Mba C. Guimaraes E. P. Ghosh K. (2012). Re-orienting crop improvement for the changing climatic conditions of the 21st century. Agric. Food Secur 1, 1–17. doi: 10.1186/2048-7010-1-7/TABLS/2

[B24] Mendiburu F. Simon R. De Mendiburu F. (2015). Ten years of an open-source statistical tool for experiments in breeding, agriculture, and biology. PeerJ Preprints doi: 10.7287/peerj.preprints.1404v1

[B25] Mir R. R. Rustgi S. Sharma S. Singh R. Goyal A. Kumar J.. (2008). A preliminary genetic analysis of fiber traits and the use of new genomic SSRs for genetic diversity in jute. Euphytica 161, 413–427. doi: 10.1007/S10681-007-9597-X/FIGURES/3

[B26] Nadarajan N. Gunasekaran M. (2005). Quantitative genetics and biometrical techniques in plant breeding (New Delhi Palve: Kalyani Publishers).

[B27] Nei M. (1972). Genetic distance between populations. Am. Nat. 106, 283–292. doi: 10.1086/282771

[B28] Palve S. M. Kumar D. Chaudhury S. K. Gupta D. (2003). Genetic variation for seed yield components in jute (Corchorus spp.). Indian J. Genet. Plant Breed. 63, 235–238.

[B29] R Development Core Team (2008). R: A language and environment for statistical computing (Vienna: R Foundation for Statistical Computing). Available at: https://www.r-project.org/ (Accessed December 18, 2023).

[B30] Roy A. Dasgupta K. Hazari S. Bhattacharya S. Das A. (2020). Heterosis and combining ability for yield and yield attributing traits in tossa jute (Corchorus olitorius L.). Curr. J. Appl. Sci. Technol. 41–49. doi: 10.9734/cjast/2020/v39i4631170

[B31] Sarkar S. De R. K. Maitra D. N. Mahapatra B. S. (2013). Maximization of quality jute seed production in India. *Technical Bulletin Series: No. 5/2013* (Barrackpore, Kolkata: Central Research Institute for Jute and Allied Fibers (ICAR), 16.

[B32] Sawarkar A. Yumnam S. Mukherjee S. (2023). Combining ability and heterosis for fiber yield, fiber quality and yield attributing traits in tossa jute (Corchorus olitorius L.) under normal and drought conditions. J. Crop Weed 19, 173–185. doi: 10.22271/09746315.2023.v19.i1.1676

[B33] Sharma H. Kumar A. Hk S. Sb C. Rt M. Lal J.. (2016). Combining ability and heterosis study for fiber yield and yield attributing characters in tossa jute (Corchorus olitorius L. Vegetos - Int. J. Plant. Res. 29, 53–58. doi: 10.4172/2229-4473.1000154

[B34] Shen Y. He X. Zu F. Huang X. Yin S. Wang L.. (2024). Development of genome-wide intron length polymorphism (ILP) markers in tea plant (*Camellia sinensis*) and related applications for genetics research. Int. J. Mol. Sci. 25, 3241. doi: 10.3390/ijms25063241 38542215 PMC10969888

[B35] Singh R. Chaudhary B. (1979). Biometrical methods in quantitative genetic analysis (New Delhi: Kalyani Publishers), 318.

[B36] Song H. Liu J. He K. Ahmad W. (2021). A comprehensive overview of jute fiber reinforced cementitious composites. Case Stud. Construction Materials 15, e00724. doi: 10.1016/J.CSCM.2021.E00724

[B37] Tareq Z. Bashar K. K. Amin R. Sarker M. D. H. Moniruzzaman M. Sarker M. S. A.. (2019). Nutritional composition of some jute genotypes as vegetabls. Int. J. Veg. Sci. 26, 506–515. doi: 10.1080/19315260.2019.1658686

[B38] Turner J. H. (1953). Combining ability and inbreeding effects. Agron. J. 45, 487–490. doi: 10.2134/agronj1953.00021962004500100008x

[B39] Zhang L. Ibrahim A. K. Niyitanga S. Zhang L. Qi J. (2019). Jute (Corchorus spp.) breeding. Adv. Plant Breed. Strategies: Ind. Food Crops 6, 85–113. doi: 10.1007/978-3-030-23265-8_4

[B40] Zhang L. Li Y. Tao A. Fang P. Qi J. (2015). Development and characterization of 1,906 EST-SSR markers from unigenes in jute (Corchorus spp.). PloS One 10, e0140861. doi: 10.1371/JOURNAL.PONE.0140861 26512891 PMC4626149

