# Supplementary material for: Increasing jute (Corchorus olitorius L.) fiber yield through hybridization and combining ability studies to break the yield plateau
Source: Front Plant Sci. 2025 Feb 26;16:1499256. doi: 10.3389/fpls.2025.1499256 (PMC11896985; doi:10.3389/fpls.2025.1499256)
Supplement: Supplementary file 1 [file Table1.docx]

Supplementary Table 1: Estimation mid parent heterosis (MPH) for fibre yield and yield components

| Entry | Plant Height | Basal Diameter | Fibre Weight | Stick Weight | Green Weight |
| --- | --- | --- | --- | --- | --- |
| JROBA 3 × JRO 2407 | 4.04 | 28.18* | 66.15 ** | 43.05 ** | 22.88 ** |
| JROBA 3 × JRO 8432 | 0.82 | 17.63 | 24.98 ** | 45.52 ** | 34.29 ** |
| JROBA 3 × JRO 524 | 8.56 | 6.82 | 7.12 | 16.61 | 11.28 * |
| JROBA 3 × S 19 | 0.81 | 14.16 | -14.01 ** | -13.48 | 2.73 |
| JROBA 3 × JROMU 1 | -0.23 | 0.25 | -4.79 | 3.1 | -7.46 |
| JROBA 3 × JBO 1 | 4.45 | 18.52 * | 16.16 * | 24.68 | 25.83 ** |
| JROBA 3 × JROBA 4 | -8.54 * | 13.81 | 16.97* | 10.21 | 7.14 |
| JROBA3 × JROM1 | -2.33 | -6.43 | 12.54 * | 59.03 * | 2.02 |
| JROBA 3 × JRO 204 | -1.47 | -2.39 | -14.78 ** | -1.13 | -14.8 |
| JRO 2407 × JROBA 3 | 0.13 | -2.33 | -8.31 ** | 11.29 ** | -9.92 |
| JRO 2407 × JRO 8432 | -0.45 | 8.57 | 28.06 ** | 48.31 ** | 25.69 ** |
| JRO 2407 × JRO 524 | 2.9 | -12.37 | 27.65 ** | 59.85 ** | 15.33 * |
| JRO 2407 × S 19 | -2.33 | -9.89 | 12.36 | -2.54 | -13.99 ** |
| JRO 2407 × JROMU 1 | -2.56 | -9.61 | 9.01 | -8.16 | -24.94 |
| JRO 2407 × JBO 1 | -1.61 | -7.72 | 37.09 ** | 33.33 ** | -16.81 |
| JRO 2407 × JROBA 4 | -13.98 ** | 1.75 | -12.38 | 9.2 | -7.83 |
| JRO 2407 × JROM 1 | 1.15 | -3.85 | 13.57 ** | 22.80 ** | -4.17 |
| JRO 2407 × JRO 204 | -0.55 | -9.80 * | 0.48 | 10.01 | 11.69 * |
| JRO 8432 × JROBA3 | -3.36 | 7.95 | 16.00 ** | 16.81 ** | -0.59 |
| JRO 8432 × JRO2407 | -1.4 | 5.24 | 44.31 ** | 45.09 ** | 10.37 * |
| JRO 8432 × JRO524 | 1.18 | -2.65 | 4.80 * | 28.02 * | 9.48 |
| JRO 8432 × S19 | -2.62 | 10.56 | 4.96 | 4.74 | -18.55 * |
| JRO 8432 × JROMU1 | -4.84 | -4.56 | 1.25 | 6.93 | 21.29 ** |
| JRO 8432 × JBO1 | -3.7 | -1.06 | 15.66 ** | 19.02 | 14.95 * |
| JRO 8432 × JROBA 4 | -14.93 ** | 2.35 | 18.51 | 32.82 | 24.11 ** |
| JRO 8432 × JROM 1 | -1.75 | 19.98 | 36.44 ** | 28.04 | 35.19 ** |
| JRO8432 × JRO 204 | -7.34 | -1.08 | 3.64 | 3.74 | -3.15 |
| JRO 524 × JROBA 3 | 3.09 | 6 | 9.39 | 2.45 | 10.14 * |
| JRO 524 × JRO 2407 | -2.68 | -12.46 | 25.83 ** | 36.36 ** | -11.55 |
| JRO 524 × JRO 8432 | 1.74 | -2.65 | 27.34 * | 28.02 * | 10.53 * |
| JRO 524 × S 19 | -5.42 | -11.57 | -14.92 | -20.26 * | -13.87 |
| JRO524 × JROMU1 | 4.96 | 21.97 * | 13.92 ** | 15.68 * | 15.64 * |
| JRO 524 × JBO 1 | 3.27 | 16.12 | 29.08 * | 47.95 ** | 23.39 * |
| JRO 524 × JROBA 4 | -9.29 ** | 17.12 | 24.67 ** | 49.05 * | 34.98 ** |
| JRO 524 × JROM 1 | 2.22 | 22.15 | 18.63 | 40.52 ** | 12.94 * |
| JRO 524 × JRO 204 | -4.05 | -0.71 | -4.61 * | -7.46 | -7.93 |
| S 19 × JROBA 3 | 2.74 | -3.69 | -17.47 ** | -14.77 | -5.51 |
| S 19 × JRO 2407 | -6.54 | -0.93 | -16.05* | -1.03 | -37.64 ** |
| S 19 × JRO 8432 | -2.22 | 10.56 | -5.96 | 3.46 | 15.65 * |
| S 19 × JRO 524 | 1.85 | 1.74 | 7.81 | 17.62 | 17.96 * |
| S 19 × JROMU 1 | -4.59 | 0.89 | -9.91 | -10.26 | -1.85 |
| S 19 × JBO 1 | -7.74 | -10.66 | -24.06* | -22.87 | -17.68 * |
| S 19 × JROBA 4 | -12.64 ** | -1.38 | -8.99 | -3.14 | 10.58 * |
| S 19 × JROM 1 | 3.93 | -3.88 | -6.83 | -4.9 | -9.42 * |
| S 19 × JRO 204 | -7.02 | -2.34 | -22.55 ** | -25.01 ** | -6.23 * |
| JROMU 1 × JROBA 3 | 4.66 | 7.1 | -8.02 | -5.73 | -4.45 |
| JROMU 1 × JRO 2407 | 1.61 | 1.27 | 7.69 | 27.15 | 0.92 |
| JROMU 1 × JRO 8432 | -6.36 | 11.8 | 13.14 | 4.85 | -16.66 * |
| JROMU 1 × JRO 524 | 4.96 | 11.55 * | 32.64 ** | 43.44 * | 26.41 ** |
| JROMU 1 × S 19 | 2.66 | 17.29 | 7.74 | 14.62 | 7.18 |
| JROMU 1 × JBO 1 | -8.52 | -6.92 | 2.03 | -13.8 | -9.54 |
| JROMU 1 × JROBA 4 | -12.04 ** | 2.08 | -13.48 | -29.01 | 8.27 |
| JROMU 1 × JROM 1 | -2.02 | -10.26 | -0.29 * | -21.03 | -16.22 ** |
| JROMU 1 × JRO 204 | -0.87 | 9.9 | 1.2 | 5.95 | 9.33 |
| JBO 1 × JROBA 3 | 3.95 | 11.83 * | 10.06 * | 4.09 | 1.7 |
| JBO 1 × JRO 2407 | 3.93 | 3.78 | 30.38 ** | 62.93 ** | 15.28 * |
| JBO 1 × JRO 8432 | -3.7 | 1.49 | 18.83 ** | 4.49 | -1.82 |
| JBO 1 × JRO 524 | 7.54 | 6.62 | 0.43 * | 13.92 ** | 25.40 * |
| JBO 1 × S 19 | 7.45 | 12.17 | 18.92 * | 42.28 | 31.45 ** |
| JBO 1 × JROMU 1 | -2.88 | -0.49 | 7.45 | 2.73 | -2.96 |
| JBO 1 × JROBA 4 | -9.43 ** | 3.99 | 9.12 | 10.42 | 7.53 |
| JBO 1 × JROM 1 | 3.25 | 2.4 | 11.07 | 26.89 * | 6.84 |
| JBO1 × JRO204 | -12.69 ** | -11.3 | -18.24 ** | -11.56 | -18.31 ** |
| JROBA 4 × JROBA 3 | -8.73 * | 2.75 | -20.15 | -25.8 | 6.73 |
| JROBA 4 × JRO 2407 | -18.28 ** | -0.31 | 6.72 | -7.03 | -23.87 * |
| JROBA 4 × JRO 8432 | -18.42 ** | -6.24 | -1.83 | -3.8 | 9.62 |
| JROBA 4 × JRO 524 | -12.72 ** | -4.91 | 10.93 ** | 6.57 * | -2.56 |
| JROBA 4 × S 19 | -17.77 ** | -10.5 | -3.76 | -4.49 | -21.88 * |
| JROBA 4 × JROMU 1 | -12.70 ** | 3.11 | 8.12 | 5.49 | 11.92 * |
| JROBA 4 × JBO1 | -9.43 ** | 10.59 | -6.99 | -6.48 | -2.12 |
| JROBA 4 × JROM1 | -19.80 ** | -3.01 | -16.29 ** | -25.74 | 10.67 |
| JROBA 4 × JRO 204 | -12.36 ** | 4.5 | -11.60 * | 0.9 | 5.25 |
| JROM 1 × JROBA 3 | 5.21 | 12.14 | 10.90 * | -8.54 * | -2.53 |
| JROM 1 × JRO 2407 | 6.29 | -2.23 | 29.66 ** | 49.81 ** | 7.41 |
| JROM 1 × JRO 8432 | -3.32 | -6.56 | 4.35 ** | -10.8 | -11.98 * |
| JROM 1 × JRO 524 | 9.56 | -0.15 | -9.45 | 29.77 ** | 6.6 |
| JROM 1 × S 19 | -1.29 | -0.55 | 15.41 | 30.59 | 11.92 * |
| JROM 1 × JROMU 1 | 5.06 | 11.51 | 29.40 * | 32.87 | 8.64 |
| JROM 1 × JBO 1 | -2.75 | -8.14 | -4.8 | 13.91 | -9.6 |
| JROM 1 × JROBA 4 | -12.46 ** | -8.98 | -16.49 ** | 1.18 | -5.53 |
| JROM 1 × JRO 204 | -8.98 | -0.72 | -17.76 ** | 7.13 | 4.75 |
| JRO 204 × JROBA 3 | -2.18 | -5.99 | -13.80 ** | -16.86 | -7.42 |
| JRO 204 × JRO 2407 | -6.97 | -19.31 * | -2.68 | -12.23 | -11.48 * |
| JRO 204 × JRO 8432 | -7.05 | -6.81 | -5.95 | -9.82 | -19.07 ** |
| JRO 204 × JRO 524 | -0.92 | -8.77 | -19.03 * | -3.77 | 7.82 |
| JRO 204 × S 19 | -6.34 | -16.61 | -34.08 ** | -30.74 ** | -38.50 * |
| JRO 204 × JROMU 1 | -7.93 | 1.2 | -15.58 | -5.5 | -14.53 * |
| JRO 204 × JBO 1 | -11.95 ** | -13.17 | -18.50 ** | -22.35 | -13.26 * |
| JRO 204 × JROBA 4 | -7.00 ** | 4.75 | -12.13 * | -5.11 | 22.47 * |
| JRO 204 × JROM 1 | -6.59 | -17.98 | -29.55 ** | -22.08 | -12.06 * |

*P<0.05, **P<0.01

Supplementary Table 2: Estimation better parent heterosis (BPH) for fibre yield and yield components

| Entry | Plant Height | Basal Diameter | Fibre Weight | Stick Weight | Green Weight |
| --- | --- | --- | --- | --- | --- |
| JROBA 3 × JRO 2407 | -0.29 | 20.74 | 39.41** | 21.37 ** | 21.08 * |
| JROBA 3 × JRO 8432 | -4.46 | 14.38 | 10.15 * | 28.85 ** | 23.76 * |
| JROBA 3 × JRO 524 | 7.16 | 1.68 | -6.72 | -0.08 | 4.7 |
| JROBA 3 × S 19 | -4.58 | 12.25 | -16.61 ** | -14.14 | 0.6 |
| JROBA 3 × JROMU 1 | -1.52 | -1.59 | -16.36 ** | -8.7 | -10.37 * |
| JROBA 3 × JBO 1 | -0.52 | 13.11 | 8.17 | 11.83* | 25.81 * |
| JROBA 3 × JROBA 4 | -20.57 ** | 11.39 | 7.4 | 2.21 | 3.14 |
| JROBA3 × JROM1 | -4.25 | -12.12 | 6.62 | 35.11** | -2.65 |
| JROBA 3 × JRO 204 | -5.91 | -10.31 | -19.36 ** | -5.12 | -17.16 * |
| JRO 2407 × JROBA 3 | -4.04 | -8 | -23.07** | -5.57 | -11.24 * |
| JRO 2407 × JRO 8432 | -1.62 | 5.08 | 20.96 ** | 41.19 ** | 14.29 * |
| JRO 2407 × JRO 524 | -0.13 | -13.33 | 22.19 ** | 57.97 ** | 7.03 |
| JRO 2407 × S 19 | -3.59 | -13.74 | -3.27 * | -17.82 | -16.98 ** |
| JRO 2407 × JROMU 1 | -5.43 | -13.33 | 3.32 | -12.57 | -28.34 * |
| JRO 2407 × JBO 1 | -2.24 | -8.98 | 22.44 ** | 25.19 ** | -18.03 * |
| JRO 2407 × JROBA 4 | -22.41 ** | -2.16 | -20.66 | -0.89 | -12.53 * |
| JRO 2407 × JROM 1 | -1.17 | -4.16 | -0.18 | 22.60 * | -9.84 |
| JRO 2407 × JRO 204 | -0.93 | -12.15 * | -19.36 ** | -9.75 * | 10.19 * |
| JRO 8432 × JROBA3 | -8.42 | 4.96 | 2.23 | 3.44 | -8.38 |
| JRO 8432 × JRO2407 | -2.56 | 1.85 | 36.31 ** | 38.12 ** | 0.35 |
| JRO 8432 × JRO524 | -2.92 | -4.75 | 3.36 | 23.27** | 7.09 |
| JRO 8432 × S19 | -2.74 | 9.31 | -4.92 | -7.86 | -23.45 * |
| JRO 8432 × JROMU1 | -8.69 * | -5.48 | 0.88 | 6.93 | 15.22 * |
| JRO 8432 × JBO1 | -4.22 | -2.95 | 8.97 | 17.31* | 5.95 |
| JRO 8432 × JROBA 4 | -22.45 ** | 1.67 | 13.29 * | 26.29** | 18.6 * |
| JRO 8432 × JROM 1 | -5.1 | 15.76 | 26.36** | 22.08** | 30.35 ** |
| JRO8432 × JRO 204 | -8.08 | -6.68 | -12.91 ** | -11.37 | -13 |
| JRO 524 × JROBA 3 | 1.76 | 0.9 | -4.74 | -12.21 | 3.63 |
| JRO 524 × JRO 2407 | -5.55 | -13.42 | 20.45 ** | 34.76 ** | -17.92 * |
| JRO 524 × JRO 8432 | -2.39 | -4.75 | 25.59** | 23.27 ** | 8.11 |
| JRO 524 × S 19 | -9.36 | -14.44 | -23.88 ** | -32.11** | -17.32 * |
| JRO524 × JROMU1 | 4.95 | 18.2 | 12.76 ** | 11.39* | 12.23 |
| JRO 524 × JBO 1 | -0.4 | 15.8 | 20.05* | 40.48 ** | 16.11 * |
| JRO 524 × JROBA 4 | -20.33 ** | 13.84 | 17.61* | 36.74 ** | 31.81 * |
| JRO 524 × JROM 1 | 1.52 | 20.43 | 8.47 | 39.09 ** | 11.29 |
| JRO 524 × JRO 204 | -7.22 | -4.34 | -20.74 ** | -23.37 * | -15.62 * |
| S 19 × JROBA 3 | -2.75 | -5.31 | -19.97 ** | -15.41 | -7.47 |
| S 19 × JRO 2407 | -7.74 | -5.16 | -27.73 ** | -16.54 | -39.81 ** |
| S 19 × JRO 8432 | -2.34 | 9.31 | -14.81 | -8.98 | 8.7 |
| S 19 × JRO 524 | -2.4 | -1.56 | -3.54 * | 0.15 | 13.24 * |
| S 19 × JROMU 1 | -8.56 | 0.72 | -18.66* | -21.05 | -2.94 |
| S 19 × JBO 1 | -8.34 | -13.33 | -27.18** | -31.28 | -19.38 ** |
| S 19 × JROBA 4 | -20.27 ** | -1.84 | -13.99 | -10.79 | 8.66 |
| S 19 × JROM 1 | 0.27 | -8.26 | -9.04 | -19.7 | -11.79 |
| S 19 × JRO 204 | -7.87 | -8.84 * | -28.80 ** | -27.51 ** | -10.67 * |
| JROMU 1 × JROBA 3 | 3.3 | 5.13 | -19.20 ** | -16.53 | -7.45 |
| JROMU 1 × JRO 2407 | -1.38 | -2.9 | 2.07 | 21.04 * | -3.64 |
| JROMU 1 × JRO 8432 | -10.15 * | 10.72 | 12.73* | 4.85 | -20.83 * |
| JROMU 1 × JRO 524 | 4.95 | 8.1 | 31.29 ** | 38.12 ** | 22.68 ** |
| JROMU 1 × S 19 | -1.61 | 17.1 | -2.72 | 0.83 | 5.98 |
| JROMU 1 × JBO 1 | -11.76 | -9.56 | -4.2 | -15.05 | -12.38 * |
| JROMU 1 × JROBA 4 | -22.74 ** | 1.77 | -17.58 | -32.5 | 7.58 |
| JROMU 1 × JROM 1 | -2.69 | -14.22 | -7.97 | -24.7 | -17.51 * |
| JROMU 1 × JRO 204 | -4.14 | 2.74 | -15.21 ** | -9.47 | 3.05 |
| JBO 1 × JROBA 3 | -1 | 6.73 | 2.49 | -6.64 | 1.69 |
| JBO 1 × JRO 2407 | 3.27 | 2.36 | 16.45 ** | 52.98 ** | 13.58 * |
| JBO 1 × JRO 8432 | -4.22 | -0.44 | 11.96* | 2.98 | -9.51 |
| JBO 1 × JRO 524 | 3.71 | 6.33 | -6.6 | 8.17 | 18 * |
| JBO 1 × S 19 | 6.75 | 8.82 | 14.04* | 26.77 ** | 28.74 ** |
| JBO 1 × JROMU 1 | -6.33 | -3.31 | 0.89 | 1.25 | -6.01 |
| JBO 1 × JROBA 4 | -17.83 ** | 1.35 | 7.47 | 6.47 | 3.52 |
| JBO 1 × JROM 1 | 0.25 | 0.68 | 9.05 | 19.33 * | 1.95 |
| JBO1 × JRO204 | -12.92 ** | -14.77 * | -27.65 ** | -23.51 ** | -20.58 * |
| JROBA 4 × JROBA 3 | -20.73 ** | 0.56 | -26.68** | -31.18 | 2.74 |
| JROBA 4 × JRO 2407 | -26.29 ** | -4.13 | -3.38 | -15.62 | -27.75 * |
| JROBA 4 × JRO 8432 | -25.63 ** | -6.86 | -6.16 | -8.53 | 4.76 |
| JROBA 4 × JRO 524 | -23.34 ** | -7.58 | 4.65 | -2.23 | -4.85 |
| JROBA 4 × S 19 | -24.96 ** | -10.92 | -9.04 | -12.03 | -23.23 * |
| JROBA 4 × JROMU 1 | -23.32 ** | 2.79 | 3 | 0.31 | 11.21 |
| JROBA 4 × JBO1 | -17.83 ** | 7.78 | -8.39 | -9.82 | -5.77 |
| JROBA 4 × JROM1 | -29.13 ** | -7.02 | -19.03 ** | -32.5 ** | 9.66 |
| JROBA 4 × JRO 204 | -20.68 ** | -2.03 | -22.81 ** | -9.89 | -1.38 |
| JROM 1 × JROBA 3 | 3.15 | 5.31 | 5.07 | -22.29 | -7 |
| JROM 1 × JRO 2407 | 3.85 | -2.54 | 13.95* | 49.56 ** | 1.05 |
| JROM 1 × JRO 8432 | -6.62 | -9.84 | -3.36 | -14.95 | -15.14 * |
| JROM 1 × JRO 524 | 8.81 | -1.55 | -17.2 | 28.45 * | 5.05 |
| JROM 1 × S 19 | -4.76 | -5.09 | 12.67* | 10.26 | 8.99 |
| JROM 1 × JROMU 1 | 4.34 | 6.59 | 19.43* | 26.68 * | 6.98 |
| JROM 1 × JBO 1 | -5.58 | -9.68 | -6.53 | 7.12 | -13.73 |
| JROM 1 × JROBA 4 | -22.65 ** | -12.74 | -19.22 ** | -8.04 | -6.39 |
| JROM 1 × JRO 204 | -11.39 * | -3.01 | -26.04 ** | -12.00 ** | -2.69 |
| JRO 204 × JROBA 3 | -6.6 | -13.62 | -18.44 ** | -20.21 | -9.98 |
| JRO 204 × JRO 2407 | -7.32 | -21.42 * | -21.89 ** | -28.00 * | -12.67 |
| JRO 204 × JRO 8432 | -7.79 | -12.09 | -20.97 ** | -22.95 | -27.3 |
| JRO 204 × JRO 524 | -4.2 | -12.11 | -32.72 ** | -20.32 * | -1.19 |
| JRO 204 × S 19 | -7.2 | -22.16 * | -39.40 ** | -33.05 ** | -41.41 * |
| JRO 204 × JROMU 1 | -10.97 | -5.4 | -29.27 ** | -19.26 | -19.44 * |
| JRO 204 × JBO 1 | -12.18 ** | -16.56 * | -27.88 ** | -32.84 ** | -15.67 |
| JRO 204 × JROBA 4 | -15.83 ** | -1.8 | -23.28 ** | -15.26 | 14.76 |
| JRO 204 × JROM 1 | -9.07 * | -19.88 | -36.64 ** | -36.00 ** | -18.3 |

*P<0.05, **P<0.01

Supplementary Table 3: Estimation standard heterosis (SH) for fibre yield and yield components

| Entry | Plant Height | Basal Diameter | Fibre Weight | Stick Weight | Green Weight |
| --- | --- | --- | --- | --- | --- |
| JROBA 3 × JRO 2407 | -1.05 | 14.43* | 24.42** | 11.58* | 17.82 * |
| JROBA 3 × JRO 8432 | -2.91 | 1.43 | -1.69 | 18.46* | 16.91 * |
| JROBA 3 × JRO 524 | 0.07 | -5.75 | -16.75 ** | -8.14 | -1.09 |
| JROBA 3 × S 19 | -2.79 | -2.71 | -25.58 ** | -19.86 * | -4.97 |
| JROBA 3 × JROMU 1 | -8.02 | -14.42 | -25.35 ** | -16.07 * | -15.34 * |
| JROBA 3 × JBO 1 | 0.01 | 4.28 | -3.46 | 2.81 | 18.85 ** |
| JROBA 3 × JROBA 4 | -1.95 | -2.53 | -4.15 ** | -6.04 * | -2.58 |
| JROBA3 × JROM1 | -9.33 | -16.18 | -4.84 | 24.21 ** | -8.04 |
| JROBA 3 × JRO 204 | -5.91 | -10.31 | -19.36 ** | -5.12 | -17.16 * |
| JRO 2407 × JROBA 3 | -4.77 | -12.8 | -31.34 | -13.19 | -13.63 * |
| JRO 2407 × JRO 8432 | -0.01 | -0.41 | -17.67 * | 0.07 | 11.21 * |
| JRO 2407 × JRO 524 | -0.89 | -17.86 * | -19.13 ** | 3.65 | 4.15 |
| JRO 2407 × S 19 | -1.79 | -18.24 * | -18.90 ** | -23.30 * | -19.21 ** |
| JRO 2407 × JROMU 1 | -6.15 | -17.86* | -30.19 ** | -38.04 ** | -30.26 * |
| JRO 2407 × JBO 1 | -1.71 | -13.74* | -5.76 | -8.63 | -20.24 * |
| JRO 2407 × JROBA 4 | -4.23 | -7.27 | -40.79 ** | -22.11 ** | -14.88 * |
| JRO 2407 × JROM 1 | -1.92 | -8.59 | -20.28 ** | -21.19 | -12.26 |
| JRO 2407 × JRO 204 | -0.93 | -12.15 * | -19.36 ** | -9.75 * | 10.19 * |
| JRO 8432 × JROBA3 | -6.93 | -6.92 | -8.76 | -4.91 | -13.46 * |
| JRO 8432 × JRO2407 | -0.97 | -3.47 | -7.22 * | -2.11 | -2.35 |
| JRO 8432 × JRO524 | -1.34 | -11.71 | -29.65 ** | -12.63 | -10.8 * |
| JRO 8432 × S19 | -0.92 | -3.07 | -20.28 ** | -14 | -30.69 * |
| JRO 8432 × JROMU1 | -7.21 | -16.18 | -31.34 ** | -24.21 ** | 1.98 |
| JRO 8432 × JBO1 | -2.66 | -10.53 | -16.13 ** | -14.39 * | 0.06 |
| JRO 8432 × JROBA 4 | -4.27 | -9.84 | -15.44 ** | -0.74 | 3.66 |
| JRO 8432 × JROM 1 | -3.56 | 10.42* | 0.92 * | -13.47 ** | 11.84 * |
| JRO8432 × JRO 204 | -6.58 | -6.68 | -12.91 ** | -11.37 | -13 |
| JRO 524 × JROBA 3 | -4.97 | -6.47 | -14.98 ** | -19.3 | -2.11 |
| JRO 524 × JRO 2407 | -6.26 | -17.94 * | -20.28 ** | -11.58 | -20.12 * |
| JRO 524 × JRO 8432 | -0.8 | -11.71 | -14.52 ** | -12.63 | -9.95 |
| JRO 524 × S 19 | -7.66 | -20.69 * | -36.18 ** | -36.63 * | -25.14 * |
| JRO524 × JROMU1 | -1.98 | 9.56* | -23.81 ** | -21.05 | -0.66 |
| JRO 524 × JBO 1 | 0.13 | 7.34* | -7.61 ** | 2.53 | 9.65 |
| JRO 524 × JROBA 4 | -1.66 | 5.52 | -12.22 ** | 7.47* | 15.2 * |
| JRO 524 × JROM 1 | -3.87 | 14.86* | -13.37 ** | -8.74 | -4.51 |
| JRO 524 × JRO 204 | -7.22 | -4.34 | -20.74 ** | -23.37 * | -15.62 * |
| S 19 × JROBA 3 | -0.93 | -17.92 | -28.57 ** | -21.05 * | -12.59 * |
| S 19 × JRO 2407 | -6.02 | -10.11 * | -39.40 ** | -22.11 * | -41.43 ** |
| S 19 × JRO 8432 | -0.51 | -3.07 | -28.57 ** | -15.05 | -1.58 |
| S 19 × JRO 524 | -0.57 | -8.75 * | -19.13 ** | -6.53 * | 2.53 |
| S 19 × JROMU 1 | -6.84 | -12.41 | -31.80 ** | -26.32 | -12.12 |
| S 19 × JBO 1 | -6.63 | -20.1 | -38.94 ** | -35.86 | -23.86 * |
| S 19 × JROBA 4 | -1.58 | -14.11 ** | -27.88 ** | -16.74 | -1.62 |
| S 19 × JROM 1 | 2.15 | -12.5 | -23.74 ** | -25.05 | -20.13 * |
| S 19 × JRO 204 | -6.15 | -8.84 * | -28.80 ** | -27.51 ** | -10.67 * |
| JROMU 1 × JROBA 3 | -3.52 | -8.57 | -27.88 ** | -23.26 * | -12.58 |
| JROMU 1 × JRO 2407 | -2.12 | -7.98 | -31.03 ** | -14.21 ** | -6.23 |
| JROMU 1 × JRO 8432 | -8.69 | -1.81 | -23.28 ** | -25.68 ** | -29.92 ** |
| JROMU 1 × JRO 524 | -1.98 | 0.21 | -11.29 ** | -2.11 | 8.59 |
| JROMU 1 × S 19 | 0.23 | 1.83 | -18.44 ** | -5.89 | -4.05 |
| JROMU 1 × JBO 1 | -11.29 | -16.62 * | -26.27 ** | -38.00 ** | -17.25 * |
| JROMU 1 × JROBA 4 | -4.64 | -10.95 | -38.48 ** | -46.95 ** | -4.77 |
| JROMU 1 × JROM 1 | -7.85 | -18.18 | -26.50 ** | -46.63 ** | -26.98 * |
| JROMU 1 × JRO 204 | -4.14 | 2.74 | -15.21 ** | -9.47 | 3.05 |
| JBO 1 × JROBA 3 | -0.46 | -1.61 | -8.53 | -14.18 | -3.94 |
| JBO 1 × JRO 2407 | 3.82 | -2.99 | -10.37 | 11.65* | 10.53 |
| JBO 1 × JRO 8432 | -2.66 | -8.22 | -13.83 ** | -24.84 * | -14.54 * |
| JBO 1 × JRO 524 | 4.27 | -1.44 | -28.11 ** | -21.05 | 11.44 * |
| JBO 1 × S 19 | 8.75 | 0.31 | -4.38 ** | 18.32* | 21.58 ** |
| JBO 1 × JROMU 1 | -5.83 | -10.86 * | -22.35 ** | -26.11 ** | -11.24 |
| JBO 1 × JROBA 4 | 1.42 | -6.57 | -17.28 ** | -16.32 * | -2.23 |
| JBO 1 × JROM 1 | 0.78 | -3.97 | -12.91 ** | -12.91 | -3.71 |
| JBO1 × JRO204 | -12.45 * | -14.77 * | -27.65 ** | -23.51 ** | -20.58 * |
| JROBA 4 × JROBA 3 | -2.15 | -12.01 | -34.57 ** | -36.74 * | -2.95 |
| JROBA 4 × JRO 2407 | -9.01 | -9.14 | -27.88 ** | -33.68 ** | -29.69 * |
| JROBA 4 × JRO 8432 | -8.2 | -17.41 | -29.96 ** | -28.11 | -8.44 |
| JROBA 4 × JRO 524 | -5.38 | -14.33 | -21.89 ** | -23.16 | -16.84 * |
| JROBA 4 × S 19 | -7.37 | -22.06 ** | -23.74 ** | -17.89 | -30.49 * |
| JROBA 4 × JROMU 1 | -5.35 | -10.06 | -23.12 ** | -21.16 ** | -1.56 |
| JROBA 4 × JBO1 | 1.42 | -0.64 | -29.50 ** | -29.12 * | -11.01 |
| JROBA 4 × JROM1 | -12.53 | -11.31 * | -35.33 ** | -46.95 ** | -4.16 |
| JROBA 4 × JRO 204 | -2.09 | -2.03 | -22.81 ** | -9.89 | -1.38 |
| JROM 1 × JROBA 3 | -2.32 | 0.45 | -6.23 | -28.56 | -12.15 |
| JROM 1 × JRO 2407 | 3.07 | -7.04 | -8.99 ** | -3.86 | -1.66 |
| JROM 1 × JRO 8432 | -5.1 | -14 | -22.81 * | -39.72 ** | -27.19 * |
| JROM 1 × JRO 524 | 3.04 | -6.1 | -33.87 ** | -15.72 | -9.87 |
| JROM 1 × S 19 | -2.98 | -9.47 | -5.53 ** | 2.91 | -1.32 |
| JROM 1 × JROMU 1 | -1.19 | 1.66 | -4.61 ** | -10.21 ** | -5.31 |
| JROM 1 × JBO 1 | -5.07 | -13.85 | -25.35 ** | -21.82 | -18.53 * |
| JROM 1 × JROBA 4 | -4.52 | -16.77 * | -35.49 ** | -27.72 ** | -18.19 * |
| JROM 1 × JRO 204 | -11.39 * | -3.01 | -26.04 ** | -12.00 ** | -2.69 |
| JRO 204 × JROBA 3 | -6.6 | -13.62 | -18.44 ** | -20.21 | -9.98 |
| JRO 204 × JRO 2407 | -7.32 | -21.42 * | -21.89 ** | -28.00 * | -12.67 |
| JRO 204 × JRO 8432 | -6.29 | -12.09 | -20.97 ** | -22.95 | -27.3 |
| JRO 204 × JRO 524 | -4.2 | -12.11 | -32.72 ** | -20.32 * | -1.19 |
| JRO 204 × S 19 | -5.46 | -22.16 * | -39.40 ** | -33.05 ** | -41.41 * |
| JRO 204 × JROMU 1 | -10.97 | -5.4 | -29.27 ** | -19.26 | -19.44 * |
| JRO 204 × JBO 1 | -11.71 * | -16.56 * | -27.88 ** | -32.84 ** | -15.67 * |
| JRO 204 × JROBA 4 | 3.89 | -1.8 | -23.28 ** | -15.26 | 14.76 * |
| JRO 204 × JROM 1 | -9.07 * | -19.88 | -36.64 ** | -36.00 ** | -18.3 * |

*P<0.05, **P<0.01
